# *Gfi1*^*Cre*^ mice have early onset progressive hearing loss and induce recombination in numerous inner ear non-hair cells

**DOI:** 10.1038/srep42079

**Published:** 2017-02-09

**Authors:** Maggie Matern, Sarath Vijayakumar, Zachary Margulies, Beatrice Milon, Yang Song, Ran Elkon, Xiaoyu Zhang, Sherri M. Jones, Ronna Hertzano

**Affiliations:** 1 Department of Otorhinolaryngology Head and Neck Surgery, University of Maryland School of Medicine, Baltimore, MD 21201, USA; 2 Department of Special Education and Communication Disorders, University of Nebraska Lincoln, Lincoln, Nebraska 68583-0738, USA; 3 Institute for Genome Sciences, University of Maryland School of Medicine, Baltimore, MD 21201, USA; 4 Department of Human Molecular Genetics and Biochemistry, Sackler School of Medicine, Tel Aviv University, Tel Aviv 69978, Israel; 5 Department of Anatomy and Neurobiology, University of Maryland School of Medicine, Baltimore, MD 21201, USA

## Abstract

Studies of developmental and functional biology largely rely on conditional expression of genes in a cell type-specific manner. Therefore, the importance of specificity and lack of inherent phenotypes for *Cre-*driver animals cannot be overemphasized. The *Gfi1*^*Cre*^ mouse is commonly used for conditional hair cell-specific gene deletion/reporter gene activation in the inner ear. Here, using immunofluorescence and flow cytometry, we show that the *Gfi1*^*Cre*^ mice produce a pattern of recombination that is not strictly limited to hair cells within the inner ear. We observe a broad expression of Cre recombinase in the *Gfi1*^*Cre*^ mouse neonatal inner ear, primarily in inner ear resident macrophages, which outnumber the hair cells. We further show that heterozygous *Gfi1*^*Cre*^ mice exhibit an early onset progressive hearing loss as compared with their wild-type littermates. Importantly, vestibular function remains intact in heterozygotes up to 10 months, the latest time point tested. Finally, we detect minor, but statistically significant, changes in expression of hair cell-enriched transcripts in the *Gfi1*^*Cre*^ heterozygous mice cochleae compared with their wild-type littermate controls. Given the broad use of the *Gfi1*^*Cre*^ mice, both for gene deletion and reporter gene activation, these data are significant and necessary for proper planning and interpretation of experiments.

Inner ear hair cells (HCs) are mechanosensitive cells responsible for sensing and transmitting information to the brain to then be interpreted as sound or head position/movement. However, HC-specific molecular analyses of both the auditory and vestibular systems in response to noise damage, ototoxic drug exposure, or genetic manipulation, have historically been limited by the heterogeneous cellular composition of the inner ear epithelia, in which HCs make up less than 2–6% of total cells in the auditory and vestibular systems, respectively (Fig. 1A)^1^. Fortunately, the development of mouse models that result in tissue and cell type-specific Cre-mediated recombination in the inner ear have allowed for controlled spatiotemporal activation or deletion of genes of interest (for a comprehensive review of Cre models in the inner ear see Cox *et al*., 2012)^2^. Overall, the most important considerations for Cre-expressing mouse models in all fields of research should be reliable cell-type specificity with no inherent effects on phenotype in at least the heterozygous state.

One commonly used HC Cre-driver in inner ear research is the *Gfi1*^*Cre*^ knock-in mouse^1^,^3^,^4^,^5^,^6^,^7^,^8^,^9^,^10^,^11^,^12^. GFI1 is a transcriptional repressor that, in the late embryonic and postnatal inner ear, is expressed in all HCs and is required for HC differentiation and survival^13^. In 2003, Wallis *et al*. first reported that *Gfi1-null* mice are profoundly deaf and have severe balance dysfunction. They further observed that the apparent inner ear dysfunction could be directly attributed to defects in both cochlear and vestibular HC development and organization, as well as cochlear HC death that occurs in a basal to apical gradient documented as early as postnatal day 0 (P0)^13^. Importantly, the *Gfi1*^+/−^ mice were reported as phenotypically indistinguishable from wild-type littermates^13^. Based on these observations, it was therefore assumed that the replacement of one copy of *Gfi1* with the coding sequence for Cre recombinase would result in HC-specific Cre expression in the inner ear, with no negative effects on hearing or balance^14^. Thus the *Gfi1*^*Cre*^ mouse was introduced in 2010, and shown to result in specific recombination in >90% of cochlear and vestibular HCs^2^,^14^. Additionally, the reported recombination pattern in the inner ear was specific to HCs. Nevertheless, data obtained from *Gfi1*^*Cre*^ mice in our laboratory, as well as recently published data^3^,^9^, have suggested that the pattern of recombination in these mice may not be specific to HCs, and that the hearing of the mice may differ from their wild-type littermate controls.

To reconcile the discrepancy between the reported and observed phenotype of this model, we have performed a comprehensive analysis of *Gfi1*^*Cre*^ mouse inner ears to assess the cell type-specificity of Cre recombinase activity, as well as the effect of *Gfi1* haploinsufficiency on hearing, vestibular function, and gene expression. In agreement with previous reports, we observe that Cre-mediated recombination is highly efficient in the HCs of both the cochlear and vestibular systems. However, we also observe broad recombination in other cells throughout the inner ear, resulting in the Cre-expressing HCs being outnumbered by Cre-expressing non-HCs. We further identify these newborn inner ear Cre-expressing non-HCs as primarily CD45 + CD11b + Gr1- immune cells, consistent with observations showing numerous resident macrophages in the adult mouse inner ear^15^,^16^,^17^,^18^,^19^. Finally, we also assess both the vestibular and auditory phenotypes of the *Gfi1*^*Cre*^ mice, and find that heterozygotes exhibit an early onset progressive hearing loss as compared with their wild-type littermates. This hearing loss cannot be attributed to the age-related hearing loss inherent to the C57BL/6 inbred mouse strain, and may be due to minor changes in gene expression that result from *Gfi1* haploinsufficiency. These results highlight the necessity of rigorous validation of Cre-driver mouse models for proper use in research. We further suggest several strategies to allow for the continued use of the *Gfi1*^*Cre*^ mice for ear research, controlling for the identified limitations.

## Results

### *Gfi1* driven Cre recombinase expression is not HC-specific in the inner ear

**Figure 1:**
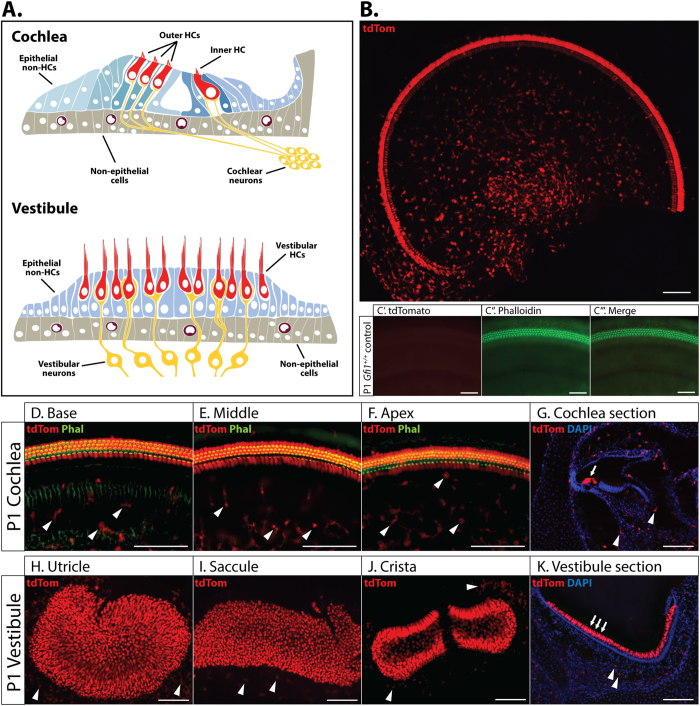
*Gfi1*^*Cre*^ mediated non-HC recombination. (**A**) Schematic of the auditory and vestibular epithelia showing a heterogeneous cellular population made up of hair cells (HCs), epithelial non-HCs, non-epithelial cells and neurons. (**B**) Whole mount immunofluorescence of an apical turn from a P1 *Gfi1*^*Cre/*+^;*ROSA26*^*CAG-tdTomato*^ mouse cochlea showing extensive non-HC recombination as a result of *Gfi1*^*Cre*^. (**C**) Cochlear whole mount immunofluorescence of a P1 *Gfi1*^+/+^;*ROSA26*^*CAG-tdTomato*^ littermate showing no recombination in any cells in the absence of Cre recombinase (n = 3). Whole mount (**D–F,H–J)** (n = 5) and section (**G,K)** (n = 3) immunohistochemistry showing the presence of non-HC *Gfi1*^*Cre*^ mediated tdTomato expressing cells (white arrowheads) in the basal, middle and apical turns of the cochlea (**D**–**F**), and utricle, saccule and crista vestibular organs (**H**–**J**). HCs are denoted by white arrows, scale bars = 100 μm.

The *Gfi1*^*Cre*^ mouse is used as a tool for inner ear HC-specific Cre recombinase expression^14^. To test the specificity of the Cre recombinase activation in the newborn mouse inner ear, we crossed the *Gfi1*^*Cre*^ mouse with the B6.Cg-*Gt*(*ROSA*)*26Sor*^*tm14(CAG-tdTomato)Hze*^/J reporter mouse (from here on referred to as *ROSA26*^*CAG-tdTomato*^). The *ROSA26*^*CAG-tdTomato*^ reporter mouse is designed to allow for robust tdTomato expression in all Cre recombinase expressing cells, as excision of a floxed stop codon within the *Rosa26* locus by Cre recombinase induces tdTomato expression under control of the endogenous ROSA26 promoter, as well as the robust CAG promoter^20^. Whole mount fluorescence microscopy of *Gfi1*^*Cre/*+^;*ROSA26*^*CAG-tdTomato*^ mice inner ears at postnatal day 1 (P1) demonstrates that *Gfi1*^*Cre*^ drives recombination in almost all HCs within the cochlear and vestibular systems. However, HCs comprise only a fraction of the total number of cells expressing tdTomato (Fig. 1B), as numerous tdTomato positive non-HCs can be observed at the basal, middle and apical regions of the cochlea, as well as within all vestibular sensory structures (Fig. 1D–F,H–J). This observation is not a result of sporadic recombination, as *Gfi1*^+/+^;*ROSA26*^*CAG-tdTomato*^ littermates show no tdTomato expression in the inner ear (Fig. 1C). Furthermore, immunohistochemistry shows that tdTomato positive non-HCs are not just limited to close proximity of the sensory epithelium, but can also be observed throughout the inner ear (Fig. 1G,K), for example in regions closer to the modiolus. This analysis demonstrates that *Gfi1*^*Cre*^-mediated recombination is not HC specific either in the cochlea or in the vestibular end organs.

### Non-HC Cre-mediated recombination is restricted to non-epithelial and non-neuronal cells

**Figure 2:**
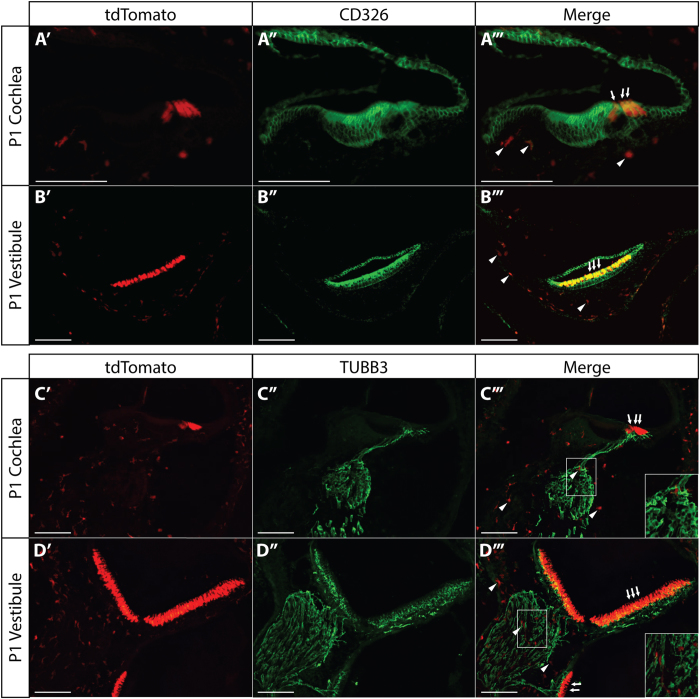
tdTomato positive non-HCs are neither epithelial nor neuronal. (**A,B**) Inner ear section immunohistochemistry from P1 *Gfi1*^*Cre/*+^;*ROSA26*^*CAG-tdTomato*^ mice stained with an antibody for the epithelial marker CD326 (EpCAM) showing no overlap of tdTomato expressing non-HCs with CD326 expression in either the auditory or the vestibular systems (n = 3). (**C,D**) P1 *Gfi1*^*Cre/*+^;*ROSA26*^*CAG-tdTomato*^ mouse inner ear sections stained with an antibody for the neuronal marker TUBB3 showing no overlap of tdTomato expressing cells with TUBB3 expression in either system (n = 3). Non-HC *Gfi1*^*Cre*^ mediated tdTomato expressing cells are denoted by white arrowheads, HCs by white arrows, scale bars = 100 μm.

It was next our goal to determine the identity of the tdTomato expressing non-HCs within the inner ears of the *Gfi1*^*Cre/*+^;*ROSA26*^*CAG-tdTomato*^ mice. It has previously been shown that all sensory and non-sensory epithelia within the inner ear express the cell surface marker CD326, also known as epithelial cell adhesion molecule (EpCAM)^21^. Cryosections from P1 *Gfi1*^*Cre/*+^;*ROSA26*^*CAG-tdTomato*^ inner ears stained with an antibody for CD326 show that the tdTomato positive HCs overlap with staining for CD326 in both the cochlea and vestibule (Fig. 2A,B). However, non-HC *Gfi1*^*Cre*^ expression is limited to non-epithelial cells, as there is no observable overlap of CD326 expression with tdTomato positive non-HCs. This shows that non-HC tdTomato positive cells are restricted to non-epithelial cells, and are thus not supporting cells.

*In situ* hybridization results from Wallis *et al*., 2003 suggest that *Gfi1*, at least at the mRNA level, may be expressed in the developing cochleovestibular ganglion (CVG) in addition to HC precursors in the otic vesicle as early as embryonic day 12.5 (E12.5)^13^. It was thus possible that Cre recombinase, under control of the *Gfi1* promoter, may be expressed in the CVG to drive the non-HC recombination. Therefore, we assessed if non-HC *Gfi1*^*Cre*^-mediated recombination could be observed in neuronal cells by staining P1 *Gfi1*^*Cre/*+^;*ROSA26*^*CAG-tdTomato*^ inner ear sections with an antibody for the neuronal marker β–tubulin class III (TUBB3). We did not observe any overlap of tdTomato and TUBB3 expression in the cochlear or vestibular ganglia (Fig. 2C,D), demonstrating that the Cre-expressing non-epithelial cells in the *Gfi1*^*Cre*^ mice are also not neuronal in nature. These results also suggest that the *Gfi1* promoter is not actively driving expression in developing CVG neurons during embryonic inner ear development.

### Cre recombinase expressing non-HCs are primarily immune cells

**Figure 3:**
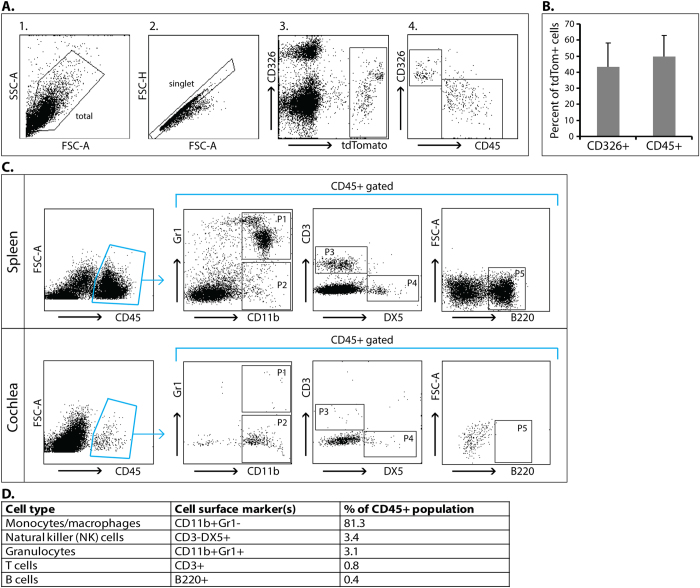
tdTomato positive inner ear cells from *Gfi1*^*Cre*^ mice consist of CD326 + CD45- cells and CD326-CD45+ cells. (**A**) Cochlear single cell suspensions from four day old *Gfi1*^*Cre/*+^*;ROSA26*^*CAG-tdTomato*^ mice were analyzed for the expression of CD326 and CD45 in the tdTomato positive cell population. One representative FACS analysis is shown. From left to right: (1) Forward and side scatter of the dissociated cells. The analysis was focused on the marked population to exclude cellular debris; (2) Gating for doublet discrimination; (3) CD326 vs tdTomato expression. The tdTomato cells (3.3% of total singlet cells) consist both of CD326 positive and negative cells; (4) Gating on the tdTomato positive cells (marked in a box in 3), the cells are divided to two distinct populations: CD326 + CD45− which represent HCs, and CD326-CD45+ which represent immune cells. (**B**) The mean + SD percentage of each population was summarized from 5 individual mice (43.4 ± 14.7% CD326 + CD45− HCs, 49.6 ± 13.2% CD326−CD45+ immune cells). (**C**) Single cell suspensions from spleen and cochleae of wild-type 4 day old C57BL/6 mice were analyzed for immune cell surface markers by FACS. 5 populations were identified within CD45+ gated cells in the cochlea: P1: 3.1% CD11b+Gr1+ granulocytes; P2: 81.3% CD11b+Gr1− monocytes/macrophages; P3: 0.8% CD3+T cells; P4: 3.4% CD3-DX5+NK cells; P5: 0.4% B220+ B cells (**D**). Data is representative of one out of two experiments using pooled cochlear cells from 5 and 7 mice, respectively. SSC-A = side scatter-A, FSC-A = forward scatter-A, FSC-H = forward scatter-H.

To quantify the non-HC tdTomato positive cells and determine their identity we next used flow cytometry. Single cell suspensions from cochleae extracted from four day old (P4) *Gfi1*^*Cre/*+^;*ROSA26*^*CAG-tdTomato*^ mice showed that the tdTomato positive cells (3.3% of total singlet cells) consisted of both CD326 positive and negative cells, consistent with the results obtained by immunohistochemistry (Fig. 3A). Because GFI1 has a known role in the immune system^22^, we further analyzed the tdTomato positive cells for expression of CD45, a leucocyte specific receptor-linked protein tyrosine phosphatase which is a commonly used broad immune cell marker^23^,^24^. We found that the tdTomato positive cells within the cochlea could be separated into two distinct populations, accounting for 93.0% of all tdTomato positive cells. These consist of CD326 + CD45- cells (43.4% of the parent population) which account for HCs, and CD326-CD45 + cells (49.6% of the parent population) which represent a population of immune cells (Fig. 3A,B). Importantly, on average, over 95% of CD45+ cells within the cochlea of the *Gfi1*^*Cre/*+^;*ROSA26*^*CAG-tdTomato*^mice were also tdTomato positive (Supplementary Figure 1).

To further define the specific types of immune cells the CD326-CD45+ tdTomato expressing cells represent, we next stained dissociated cochlear cells from P4 C57BL/6 mice with CD45 and additional canonical immune markers for T cells (CD3+), B cells (B220+), natural killer (NK) cells (CD3-DX5+), monocytes/macrophages (CD11b + Gr1−) and granulocytes (CD11b + Gr1+)^23^. Staining of a single cell suspension obtained from the spleen of the same mice was used as a positive control. Our analysis revealed the identity of 89% of the CD45+ cells in the early postnatal mouse inner ear. Specifically, the CD45+ cells consisted primarily of monocytes/macrophages (81.3%) followed by NK cells (3.4%), and a combination of granulocytes, T-cell and B-cells (4.3%) (Fig. 3C,D). These data show that, in the *Gfi1*^*Cre*^ mice, recombination occurs in all HCs as well as in CD45+ cells, most of which consist of monocytes/macrophages.

### *Gfi1* ^*Cre*^ heterozygotes display no vestibular defects

**Figure 4:**
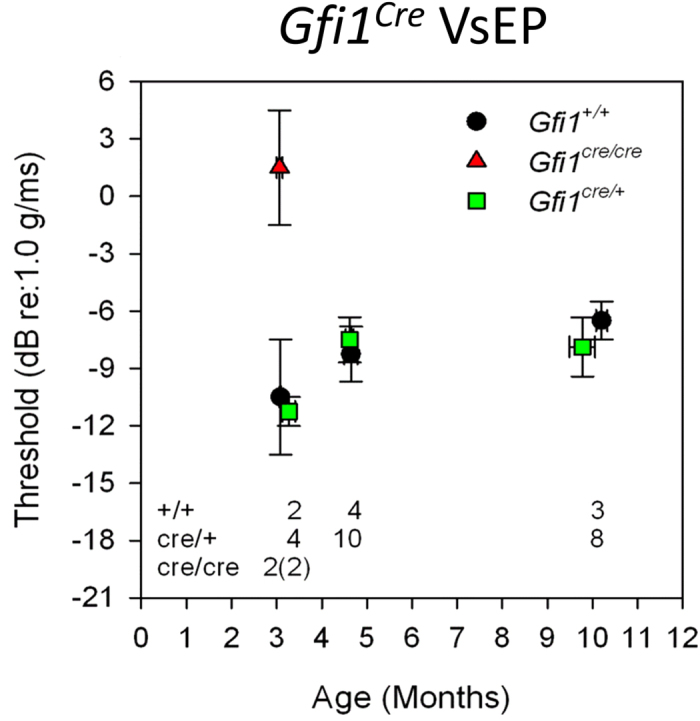
*Gfi1*^*Cre/*+^ mice do not exhibit vestibular dysfunction up to 10 months of age. Vestibular function of *Gfi1*^+/+^ (+/+), *Gfi1*^*Cre/*+^ (cre/+) and *Gfi1*^*Cre/Cre*^ (cre/cre) littermates was measured by vestibular sensory-evoked potentials (VsEP) at 3, 5 and 10 months of age. Two of the four *Gfi1*^*Cre/Cre*^ animals (number shown in parentheses) had absent responses at 3 months. Average threshold shown for *Gfi1*^*Cre/Cre*^ reflects only those animals with measurable responses (n = 2). Analysis of variance showed no significant differences in VsEP thresholds between *Gfi1*^+/+^ and *Gfi1*^*Cre/*+^ at all ages tested (*Gfi1*^+/+^ n = 2, 4, and 3 mice at 3, 5 and 10 months respectively, *Gfi1*^*Cre/*+^ n = 4, 10, and 8 mice at 3, 5 and 10 months respectively).

It has been assumed that *Gfi1*^*Cre/*+^ mice are phenotypically normal despite having only one functional copy of *Gfi1*, and can thus be a good model for investigating the effect of HC-specific Cre-mediated knockout of genes of interest^14^. Here we assessed vestibular function of *Gfi1*^*Cre/Cre*^, *Gfi1*^*Cre/*+^ and *Gfi1*^+/+^ littermates at three ages using vestibular sensory-evoked potentials (VsEP). As expected, *Gfi1*^*Cre/Cre*^ mice showed significantly elevated VsEPs at 3 months due to the loss of both functional copies of the *Gfi1* coding sequence (Fig. 4). However, *Gfi1*^*Cre/*+^ show no change in vestibular function as compared to *Gfi1*^+/+^ littermates at ages 3 and 5 months. Importantly, vestibular function does not decline up to 10 months of age in the *Gfi1*^*Cre/*+^ mice, indicating that they have normal vestibular HC development and function, and can thus be considered a good model for studying the vestibular system from embryonic to adult ages.

### *Gfi1* ^*Cre*^ heterozygotes display an early onset progressive hearing loss

**Figure 5:**
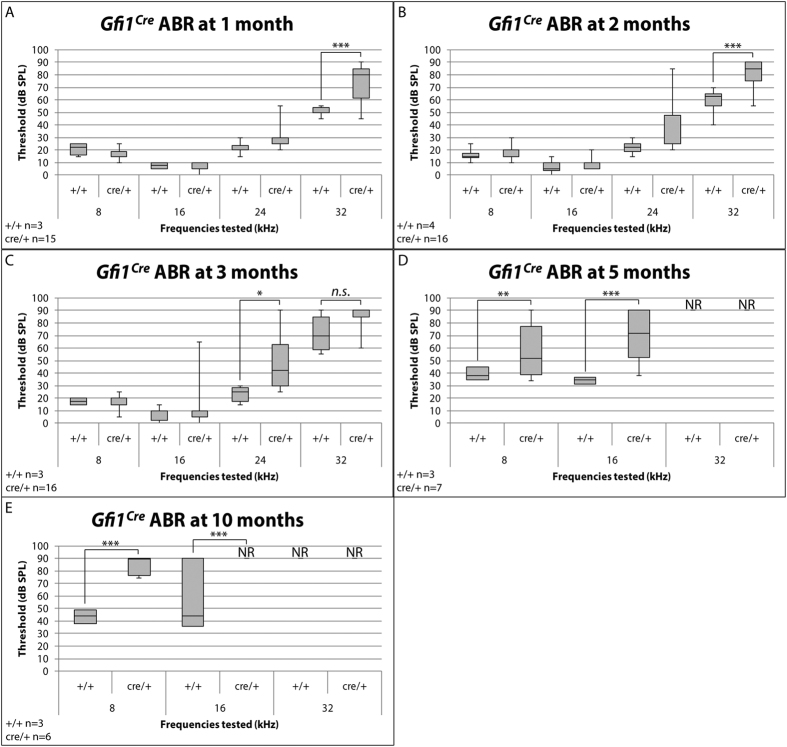
*Gfi1*^*Cre*^ heterozygotes exhibit an early onset progressive hearing loss. *Gfi1*^*Cre/Cre*^ mice showed absent ABR thresholds (>90 dB SPL, highest stimulus tested) at all frequencies and time points (up to 3 months). **(A)** Elevated hearing thresholds can be seen in one month old *Gfi1*^*Cre/*+^ mice (cre/+) as compared to *Gfi1*^+/+^ (+/+) littermates at 32 kHz (*p*-value = 6.40E-05). (**B**) High frequency hearing loss worsens as heterozygous mice age, as *Gfi1*^*Cre/*+^ mice have more pronounced elevated hearing thresholds compared to *Gfi1*^+/+^ at 32 kHz at 2 months (*p*-value = 2.72E-06). (**C**) At 3 months of age, *Gfi1*^*Cre/*+^ mice now show significantly elevated hearing thresholds at 24 kHz as compared to *Gfi1*^+/+^ littermates (*p*-value = 0.029), however both *Gfi1*^*Cre/*+^ and *Gfi1*^+/+^ mice show elevated hearing thresholds at 32 kHz (*p*-value = 0.076). (**D**) At 5 months old, *Gfi1*^*Cre/*+^ mice show significantly elevated hearing thresholds at both 8 kHz and16 kHz as compared to *Gfi1*^+/+^ littermates (*p*-value = 6.64E-03 and 7.11E-05, respectively), and (**C**) at 10 months old, *Gfi1*^*Cre/*+^ mice still show significantly elevated hearing thresholds at 8 kHz and 16 kHz as compared to *Gfi1*^+/+^ littermates (*p*-value = 3.37E-13 and 3.86E-04, respectively). *Note: at 10 months, one *Gfi1*^+/+^ mouse exhibited no response to sound stimuli at 16 kHz, while others showed average thresholds of 40.1 dB SPL. We believe that this mouse is an outlier, based on known hearing phenotypes of aged wild-type mice, but was still included in this analysis. Animal numbers: 1 month *Gfi1*^+/+^ n = 3, *Gfi1*^*Cre/*+^ n = 15; 2 months *Gfi1*^+/+^ n = 4, *Gfi1*^*Cre/*+^ n = 16; 3 months *Gfi1*^+/+^ n = 3, *Gfi1*^*Cre/*+^ n = 16; 5 months *Gfi1*^+/+^ n = 3, *Gfi1*^*Cre/*+^ n = 7; 10 months *Gfi1*^+/+^ n = 3, *Gfi1*^*Cre/*+^ n = 6. NR = no response (>90 dB SPL), *n.s.* =not significant, * = *p*-value < 0.05, **=*p*-value < 0.01, ***=*p*-value < 0.001. Significance was assessed by two-way ANOVA, followed by Tukey’s test to account for multiple comparisons, and data displayed as box plots to show variability in hearing phenotypes in *Gfi1*^*Cre/*+^ mice.

After observing that *Gfi1*^*Cre/*+^ mice do not display any vestibular dysfunction, we next assessed auditory function by measuring auditory brainstem responses (ABR). As was expected, *Gfi1*^*Cre/Cre*^ mice were profoundly deaf (ABR thresholds are greater than 90 dB SPL, highest stimulus tested) at all time points, consistent with the cochlear HC dysfunction and death observed in *Gfi1*^−/−^ mice^13^. However, in contrast to the vestibular system, where *Gfi1*^*Cre/*+^ VsEP thresholds were indistinguishable from wild-type C57BL/6J littermates up to 10 months of age, *Gfi1*^*Cre/*+^ mice show significantly elevated hearing thresholds as compared to wild-type mice at as early as 1 month of age at 32 kHz (average thresholds = 73.2 dB SPL vs. 50.8 dB SPL, *p*-value = 6.40E-05) (Fig. 5A). This high frequency hearing loss is also apparent at 2 months, where *Gfi1*^*Cre/*+^ mice continue to have elevated hearing thresholds compared to wild-type at 32 kHz (average thresholds = 79.5 dB SPL, vs. 57.5 dB SPL, *p*-value = 2.72E-06) (Fig. 5B), and by 3 months of age, hearing loss progresses in *Gfi1*^*Cre/*+^ mice to show significantly elevated hearing thresholds at 24 kHz as compared to their wild-type littermates (average thresholds = 47.0 dB SPL vs. 23.3 dB SPL, *p*-value = 0.029). Interestingly, at 3 months, both the *Gfi1*^*Cre/*+^ and *Gfi1*^+/+^ mice show elevated hearing thresholds at 32 kHz (average thresholds = 85.2 dB SPL and 71.7 dB SPL respectively, *p*-value = 0.076) (Fig. 5C), and we hypothesize that this is the consequence of a strain-specific age-related high frequency hearing loss.

Next, to determine the long-term effects of *Gfi1* haploinsufficiency on hearing, we further investigated hearing thresholds of the *Gfi1*^*Cre*^ mice at 8, 16 and 32 kHz at 5 and 10 months of age. We found that by 5 months of age, hearing thresholds are significantly elevated in *Gfi1*^*Cre/*+^ mice when compared to wild-type at 16 kHz (average thresholds = 69.5 dB SPL and 34.1 dB SPL respectively, *p*-value = 7.11E-05), and even at 8 kHz (the lowest frequency tested, average thresholds = 56.5 dB SPL and 39.1 dB SPL respectively, *p*-value = 6.64E-03), while both genotypes still show elevated hearing thresholds at 32 kHz (over 90 dB) (Fig. 5D). By 10 months, *Gfi1*^*Cre/*+^ mice have progressed to near deafness at all frequencies, with absence of measurable hearing thresholds at 16 and 32 kHz, and average thresholds of 84.9 dB SPL at 8 kHz (Fig. 5E). These results further indicate that substitution of one copy of *Gfi1* with Cre recombinase causes a progressive age related hearing loss in the *Gfi1*^*Cre/*+^ mice.

Importantly, a single nucleotide polymorphism (SNP) in *Cdh23* (753G > A, also called *Ahl*), which encodes for the essential HC tip link component Cadherin 23, has been previously reported to cause an increased susceptibility to both noise-induced and age-related hearing loss in several inbred mice strains, including C57BL/6J^25^,^26^,^27^,^28^,^29^. As the *Gfi1*^*Cre*^ mouse line was first developed on a mixed 129S6 and C57BL/6J background^14^, we wanted to ensure that the age-related hearing loss phenotype observed in *Gfi1*^*Cre*^ heterozygotes compared to wild-type littermates was not a result of a skewed distribution of *Cdh23*^753A^ genotypes within our tested population. Therefore, we genotyped each mouse at position 753 in the *Cdh23* gene by Sanger sequencing. We found that 100% of mice (*Gfi1*^+/+^, *Gfi1*^*Cre/*+^ and *Gfi1*^*Cre/Cre*^) used for ABR testing were homozygous for the *Cdh23*^753A^ allele, and are thus more susceptible to age-related hearing loss. However, this further indicates that the more severe age-related hearing loss phenotype seen in *Gfi1*^*Cre*^ heterozygotes as compared to their wild-type littermates is a result of the replacement of one copy of *Gfi1* with Cre recombinase and not the result of the *Cdh23*^753A^ allele, as all mice are homozygous for *Ahl*.

### Minimal differences in cochlear gene expression at P8 between *Gfi1* ^*Cre/*+^ and *Gfi1* ^+/+^

GFI1 is a transcription factor that, in addition to its functions in other tissues, regulates gene expression in inner ear HCs^13^,^22^,^30^,^31^. To determine if the *Gfi1*^*Cre/*+^ mice have statistically significant changes in cochlear gene expression that could contribute to their hearing loss phenotype, we extracted RNA from cochlear ducts of eight day old (P8) *Gfi1*^*Cre/*+^ and *Gfi1*^+/+^ mice and measured gene expression using RNA-seq. We chose this time point both to identify early changes resulting from *Gfi1* haploinsufficiency, as well as to eliminate a bias in gene expression that could result from a later loss of HCs. Our analysis detected 14,866 genes as expressed in the cochleae of the heterozygous and wild-type mice. Notably, differences in expression profiles between *Gfi1*^*Cre/*+^ and *Gfi1*^+/+^ mice were minimal. Only a very small number of genes (11) could be considered differentially expressed between the two groups based on standard criteria (fold change >2.0, *p*-value < 0.05; Table 1). Moreover, only 4 of these 11 genes remained statistically significant after taking into account multiple testing (false discovery rate, FDR = 0.05). Of note, these 4 genes are located on the Y chromosome, indicating that our *Gfi1*^*Cre/*+^ samples were obtained mainly from male pups, while our *Gfi1*^+/+^ population mainly from female, and thus the changes in their expression are likely unrelated to the *Gfi1*^*Cre/*+^ genotype. Levels of *Gfi1* mRNA were not significantly downregulated in the RNA-seq samples from *Gfi1*^*Cre/*+^ cochleae (fold change = 0.88). This is not surprising as only a portion of exon 1, as well as exons 2–5, are replaced by Cre recombinase, and the rest of the transcript, while not functional, will add to the overall read-counts for the gene. As the RNA-seq was performed on whole cochleae, there were not sufficient read counts to compare exon-specific transcript levels. We therefore extracted RNA from newborn *Gfi1*^*Cre/*+^ and *Gfi1*^+/+^ cochleae and measured the relative transcript abundance of exons 2–3, 5–6 and 6–7 by RT-qPCR. We detected a statistically significant 30–40% decrease in transcript abundance of the exons replaced by Cre recombinase (exons 2–3: fold change = 0.675, *p*-value = 0.022 and exons 5–6: fold change = 0.621, *p*-value = 0.005), while no change in transcript levels of exons present in both genotypes was observed (exons 6–7: fold change = 1.099, *p*-value = 0.52) (Supplementary Figure 2).

**Figure 6:**
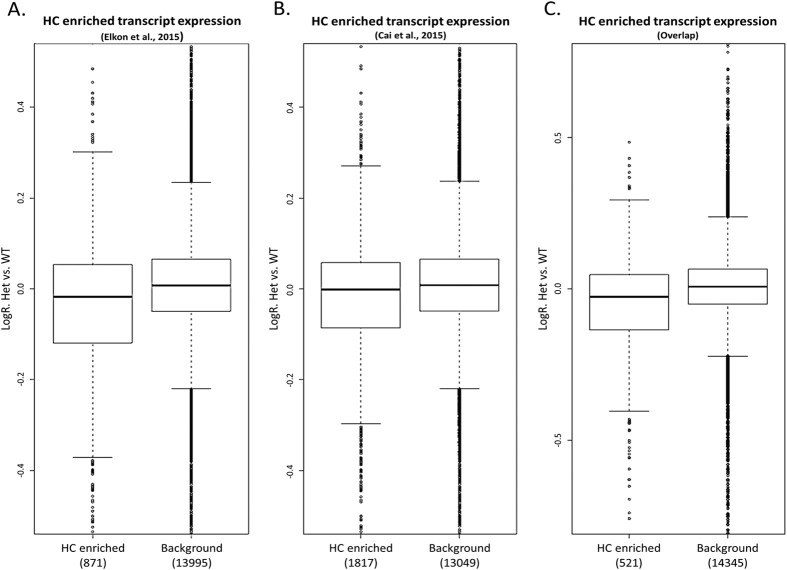
*Gfi1*^*Cre/*+^ cochleae display slight downregulation of HC-enriched transcripts. The distribution of relative gene expression levels (fold change values in log2 scale) between *Gfi1*^*Cre/*+^ (Het) and *Gfi1*^+/+^ (WT) samples was calculated for sets of HC-enriched transcripts and compared with the fold change of remaining genes in the dataset (background). HC-enriched sets of genes (consisting of genes that showed at least 3-fold elevated expression in HCs compared to non-HCs from either the (**A**) Elkon *et al*. dataset (871 genes), (**B**) Cai *et al*. dataset (1,817 genes) or (**C**) defined by the overlap between these two datasets (521 genes)), exhibited statistically significant decreased expression in *Gfi1*^*Cre/*+^ P8 cochlea compared to their wild-type control (*p*-values = (**A**) 1.67E-15, (**B**) 6.03E-11, (**C**) 1.17E-14, calculated using Wilcoxon’s test).

To further investigate this dataset, and specifically assess subtle changes in gene expression in the HCs of the *Gfi1*^*Cre/*+^ mice, we defined a group HC-enriched transcripts using two publicly available transcriptomic inner ear datasets. We identified 871 and 1,817 HC-enriched transcripts (showing at least 3-fold enriched expression in HCs compared to non-HCs) in the datasets of Elkon *et al*.^1^ and Cai *et al*.,^32^ respectively, with an overlap of 521 genes (see Supplementary Table 1). Testing these sets of genes as a whole, we observed that their expression was slightly, yet statistically significantly, decreased in *Gfi1*^*Cre/*+^ compared to *Gfi1*^+/+^ (Fig. 6A–C). Finally, as we observed a broad expression of Cre recombinase in inner ear resident macrophages in the *Gfi1*^*Cre*^mouse, we next sought to exclude the possibility that the decrease in expression of HC-enriched transcripts found in the *Gfi1*^*Cre/*+^ cochleae came from Cre positive immune cells rather than from HCs. To this end, we analyzed two publicly available RNA-seq datasets that recorded gene expression profiles in bone-marrow derived mouse macrophages (see methods)^33^,^34^. First, we confirmed that the set of HC-enriched transcripts used in the above analysis is significantly enriched in HCs also when compared to macrophages (Supplementary Figure 3A). Secondly, we used the macrophage datasets to define a set macrophage-enriched genes (Supplementary Table 3). This set contained 360 genes that showed, in both macrophage datasets, at least 3-fold enriched expression in macrophages compared to other key cell types in the inner ear. Reassuringly, Gene-Ontology (GO) functional enrichment analysis demonstrated that this set of macrophage expressed genes was significantly enriched for immune-system related functions (Supplementary Table 4). Importantly, the expression of this set of transcripts was not decreased in *Gfi1*^*Cre/*+^ compared to *Gfi1*^+/+^ (it was even slightly increased; Supplementary Figure 3B). Taken together, these results show that the decreased expression of HC-enriched transcripts observed in *Gfi1*^*Cre/*+^ cochlea compared to *Gfi1*^+/+^ can be attributed to reduced expression specifically in HCs.

## Discussion

Researchers are dependent on Cre mouse models with reliable cell-type specificity and the absence of inherent phenotypes to make accurate and informative conclusions. For this reason, it is important to comprehensively validate such models to ensure these two assumptions are met, or if they are not met, that appropriate controls and considerations be used to make the model useful. Here we have performed such a validation on the *Gfi1*^*Cre*^ mouse, a model commonly used in inner ear research for HC-specific Cre-mediated recombination^1^,^3^,^4^,^5^,^6^,^7^,^8^,^9^,^10^,^11^,^12^. Upon investigation of this mouse, we have shown that in addition to HCs, recombination is also present in a multitude of CD45+ monocytes/macrophages in the inner ear that outnumber Cre-expressing HCs. This observation indicates that *Gfi1*^*Cre*^ was expressed at one point in the development of the observed CD45+ cells, but does not necessarily mean that the gene is expressed at the time the tissue was analyzed, a common limitation of Cre-reporter mice that are not based on inducible expression. Additionally, *Gfi1*^*Cre*^ mediated recombination in CD45+ cells is not altogether unexpected, as GFI1 is highly characterized for its involvement in the development of hematopoietic cell lineages, including macrophages^35^,^36^,^37^,^38^. Although our data show that *Gfi1*^*Cre*^ does not result in HC-specific recombination, it introduces this mouse as a potential dual reporter for studying the role of HCs and resident macrophages in the inner ear (for example, for observing macrophage migration in response to noise or ototoxic drug induced damage). We have additionally observed that *Gfi1*^*Cre*^ positive cells that are not HCs are also non-epithelial. This observation provides a functional solution for the continued use of *Gfi1*^*Cre*^ mice to drive reporter gene expression for HC sorting by flow cytometry, through either co-staining with the epithelial marker CD326 (Fig. 2A), or by isolating epithelial cells by delamination before dissociation^3^. Furthermore, because the number of tdTomato positive immune cells seems to increase in a gradient from the organ of Corti towards the modiolus (Fig. 1B), it may also be possible to better exclude macrophages from HCs with altered dissection approaches.

The difference in recombination patterns seen between our Cre expression results and the original description of the *Gfi1*^*Cre*^ mice is likely the result of the reporter gene used. Previous reports of Cre model recombination patterns, such as those performed in Madisen *et al*., 2010, have shown that the use of different reporter mice can result in different recombination patterns^20^. Whether this indicates that different reporters are more or less susceptible to recombination depending on the level of Cre expression in the tissue of interest, or that differences in expression of reporter molecules after recombination can account for these discrepancies, is not precisely known, and could vary between Cre and reporter models. In the original description of the *Gfi1*^*Cre*^ mouse, the induction of Cre recombinase was tracked using an enzymatic reaction for LacZ expression under control of the ROSA26 promoter (R26R-LacZ, Jackson Laboratory stock #003310)^14^. However, the *ROSA26*^*CAG-tdTomato*^ mouse used in this study is a much more robust reporter mouse. tdTomato is a bright fluorescent molecule, and its expression is aided by the strong CAG promoter in the presence of Cre recombinase, as well as by a woodchuck hepatitis virus posttranscriptional regulatory element (WPRE) to help increase tdTomato mRNA stability^20^. Indeed, addition of the CAG promoter to drive expression in the *Rosa26* locus has been shown to result in ~9 fold higher expression than the endogenous ROSA26 promoter alone^39^.

The *Gfi1*^+/−^ mice were originally described as phenotypically indistinguishable from wild-type littermates, suggesting that knock-in of Cre recombinase would result in an inconsequential HC-specific recombination. However, this observation was based on behavioral assessment rather than quantitative neurophysiological testing^13^. Here we have shown that while in the heterozygous state *Gfi1*^*Cre*^ mice exhibit no vestibular defects, they do present with an early onset progressive hearing loss phenotype. This finding potentially undermines past and future phenotypic characterizations of conditional knockout models that utilize the *Gfi1*^*Cre*^ mouse. However, the hearing loss in *Gfi1*^*Cre/*+^ mice does not significantly affect the mice at 8 and 16 kHz up to 3 months of age. Therefore, continued use of the *Gfi1*^*Cre*^ mouse for conditional gene deletion and evaluation of hearing is possible, but necessitates concomitant analysis of *Gfi1*^*Cre/*+^ mice as controls instead of commonly used Cre-negative controls.

From previous research, we are aware that the GFI1 transcription factor plays an essential role in HC development and survival^13^,^30^,^31^,^40^. It would therefore not be surprising that replacement of one copy of *Gfi1* with the coding sequence for Cre recombinase could cause minor changes in the HC transcriptome that may eventually lead to HC dysfunction. Indeed, although our RNA-seq results showed only a few number of genes to be significantly differentially expressed early in postnatal life (all likely secondary to an uneven distribution of male and female animals used for the RNA-seq), HC-enriched transcripts were found to be overall slightly downregulated in *Gfi1*^*Cre*^ heterozygotes. Moreover, functional *Gfi1* transcript abundance as measured in newborn *Gfi1*^*Cre/*+^ cochleae by RT-qPCR was decreased by 30–40%, consistent with a possible gene-dosage effect. Alternatively, there is also the possibility that prolonged *Gfi1* driven Cre recombinase expression leads to what is termed “Cre toxicity,” which could be affecting either the function or survival of HCs in *Gfi1*^*Cre*^ heterozygote animals. Cre toxicity is a phenomenon in which prolonged Cre exposure can lead to non-specific recombination at cryptic loxP sites in the genome of Cre-expressing cells, and has been shown to lead to dose dependent cell damage and death in select Cre-expressing mouse models^41^,^42^,^43^,^44^,^45^,^46^. Although this may not pose much of a problem for studies done utilizing non-inducible Cre models early in life, it may be a challenge for studies focusing on mature animals with constitutive expression of Cre recombinase. However, if Cre toxicity is the mechanism by which *Gfi1* driven Cre recombinase expression results in cochlear HC dysfunction, the lack of a vestibular phenotype in *Gfi1*^*Cre*^ heterozygous mice would still be surprising. Published transcriptome data demonstrates that *Gfi1* expression is higher in vestibular HCs as compared to cochlear HCs, at least early in life^1^,^47^. Thus, theoretically, vestibular HCs would have increased exposure to Cre recombinase as compared to cochlear HCs, and would be more likely to accrue damage over time. It is also possible that cochlear HCs are simply more sensitive to damage resulting from Cre exposure, and thus more prone to dysfunction compared with vestibular HCs. To resolve this, a more comprehensive analysis involving several HC-expressed Cre-drivers would be necessary.

A recent study published by Walters *et al*. provided the research community with valuable insight on the use of the *Sox2*^*CreER*^ mouse model in cochlear supporting cell fate-mapping experiments. In this study, they showed that while Cre induction by tamoxifen injection at all ages resulted in highly efficient recombination in cochlear supporting cells (>85%), induction at early postnatal ages (P1) also resulted in recombination in cochlear HCs (>50%)^48^. Without this information, studies using the *Sox2*^*CreER*^ mice for testing regenerative therapies at postnatal ages could have led to inaccurate conclusions. These types of observations further accentuate the need for appropriate and in-depth validation of animal models used for research purposes. Here we have shown that the *Gfi1*^*Cre*^ mouse can still be used as a valuable tool for both conditional gene deletion and inner ear HC isolation for downstream gene expression analysis, provided that experiments are adjusted to account for non-HC Cre mediated recombination and an early onset progressive hearing loss phenotype. Of note, review of studies published to date that utilize the *Gfi1*^*Cre*^ mouse for conditional deletion/activation of target genes followed by phenotyping after one month of age did not reveal any significant conclusions that, in our opinion, could have been solely attributed to the inherent phenotype of the *Gfi1*^*Cre*^ mouse described here^5^,^6^,^7^,^8^,^11^,^12^. For all Cre models, patterns of recombination should be characterized using robust reporters, such as the *ROSA26*^*CAG-tdTomato*^ mouse (where both the CAG and endogenous ROSA26 promoters drive tdTomato expression following recombination). Finally, the presence of inherent phenotypes should be rigorously examined in heterozygous animals, and if found, it is our suggestion that heterozygous animals be included as controls in conditional gene deletion experiments instead of Cre-negative controls.

## Material and Methods

### Animals

The *Gfi1*^*Cre*^ knock-in mice were generated by Dr. Lin Gan at the University of Rochester and were generously provided for this study by Dr. Jian Zuo of the Developmental Neurobiology Department at St. Jude Children’s Research Hospital. *Gfi1*^*Cre*^ mice were maintained in a C57BL/6J background at the University of Maryland School of Medicine. B6.Cg-*Gt(ROSA)26Sor*^*tm14(CAG-tdTomato)Hze*^/J mice were procured from the Jackson Laboratory (stock #007914, Bar Harbor, ME). All procedures involving animals were carried out in accordance with the National Institutes of Health Guide for the Care and Use of Laboratory Animals and have been approved by the Institutional Animal Care and Use Committee at the University of Maryland, Baltimore (protocol numbers 1209008 and 1015003).

### Genotyping

As reported in Yang *et al., Gfi1*^*Cre*^ drives recombination in a number of other tissues in addition to the inner ear, including the liver, lung, heart, kidney, gut and pancreas^13^,^14^. For this reason, *Gfi1*^*Cre/*+^;*ROSA26*^*CAG-tdTomato*^ and *Gfi1*^+/+^;*ROSA26*^*CAG-tdTomato*^ mice were genotyped by physical observation of tdTomato red fluorescence in the liver following inner ear dissection at P1. For *Gfi1*^*Cre*^ mice used in auditory and vestibular phenotyping, genotyping was performed by polymerase chain reaction (PCR) on genomic DNA extracted from tail snips or ear punches using a modified one step protocol outlined in Yang *et al*., 2010^14^. Primers used for *Gfi1*^*Cre*^ genotyping are as follows: *Gfi1* wild-type forward (5′-GGG ATA ACG GAC CAG TTG-3′), *Gfi1* wild-type reverse (5′-CCG AGG GGC GTT AGG ATA-3′), *Gfi1*^*Cre*^ reverse (5′-GCC CAA ATG TTG CTG GAT AGT-3′). Additionally, because *Gfi1*^*Cre*^ mice were developed on a mixed 129S6 and C57BL/6J background, the *Gfi1*^*Cre*^ mice used for auditory phenotyping by ABR were also genotyped for age-related high frequency hearing loss susceptibility as denoted by polymorphisms at position 753 in the *Cdh23* gene. Genotyping was performed by PCR using the primers *Cdh23* forward (5′-GGC CAT CAT CAT CAC GGA CA-3′) and *Cdh23* reverse (5′-TAG CCC ATT TGA CCA GGT GC-3′) followed by Sanger sequencing at the University of Maryland, Baltimore Genomics Core Facility (Baltimore, MD). Genotypes for each mouse at position 753 were manually curated by scanning chromatograms in Sequence Scanner Software 2 (Applied Biosystems, Foster City, CA).

### Immunofluorescence and image acquisition

*Gfi1*^*Cre/*+^;*ROSA26*^*CAG-tdTomato*^ inner ears were dissected at P1 and fixed in 4% paraformaldehyde (PFA) in PBS overnight at 4 °C. After fixation, ears were then either stored in PBS for further dissection and whole mount immunohistochemistry or processed and embedded in OCT compound (Tissue-Tek) for cryosectioning. Immunofluorescence was determined using FITC rat anti-mouse CD326 (BioLegend, at 1:50) and mouse anti-Tubulin β 3 (TUBB3) (BioLegend, 1:500) primary antibodies. Chicken anti-mouse IgG (H + L) Alexa Fluor 488 (Invitrogen, 1:800) was used for secondary detection, Alexa Fluor 488 Phalloidin (Invitrogen, 1:500) to stain the actin cytoskeleton, and 4′,6-Diamidino-2-Phenylindole Dihydrochloride (DAPI, 1:20,000, Life Technologies) to mark the cell nuclei. Images were acquired from N = 3 *Gfi1*^*Cre/*+^;*ROSA26*^*CAG-tdTomato*^ mice for cryosection staining and N = 5 *Gfi1*^*Cre/*+^;*ROSA26*^*CAG-tdTomato*^ mice for whole mount staining using a Nikon Eclipse E600 microscope (Nikon, Tokyo, Japan) equipped with a Lumenera Infinity 3 camera and processed using Infinity Capture and Infinity Analyze software (Lumenera, Ottawa, ON).

### Flow Cytometry

Cochlear tissue dissociation was performed as previously described^21^. Briefly, the auditory sensory epithelia were collected and placed in 0.5 mg ml^−1^ Thermolysin (Sigma). Tissues were then incubated for 20 min at 37 °C, and Thermolysin was replaced with Accutase enzyme cell detachment medium (eBioscience). Tissues were incubated for 3 min at 37 °C, followed by mechanical disruption with a 23G blunt ended needle and 1 ml syringe. Tissues were incubated for an additional 3 min in Accutase, and mechanical disruption was performed a second time. Upon visual confirmation of successful dissociation, the reaction was stopped by adding an equal volume of IMDM supplemented with 10% heat-inactivated fetal bovine serum (FBS). Cells were then passed through a 40-μm cell strainer (BD) and placed on ice. The following anti-mouse monoclonal antibodies (mAbs) were used for FACS staining: CD45-FITC, CD326-APC, CD3e-Alexa Fluor 647, and Gr1-APC (obtained from BD Biosciences); B220-PerCP, CD11b-PE, and DX5-PE (obtained from BioLegend). Cochleae and spleen single cell suspensions from P4 mice were pre-treated with anti-mouse CD16/CD32 for Fc receptor blocking, followed by staining with antibodies in FACS buffer (PBS, 2% FBS, 0.05% NaN3) for 30 minutes at 4 °C. Cells were then washed and acquired by a BD LSRII flow cytometer. Data was analyzed with BD FACS Diva software (BD Biosciences, San Jose, CA). Cells were gated based on forward scatter (FSC) vs side scatter (SSC), doublets were excluded based on the forward scatter-H (FSC-H) vs forward scatter-A (FSC-A). CD45 positive (CD45+) staining defines immune cells. Within the CD45+ gate, specific immune cell populations were identified as: CD3+ T cells, B220+ B cells, CD3-DX5+NK cells, CD11b+Gr1− monocytes/macrophages, and CD11b+Gr1+ granulocytes.

### Vestibular evoked potentials (VsEPs)

For VsEP testing, mice were anesthetized with a ketamine (90–100 mg/kg) and xylazine (10–12 mg/kg) solution injected intraperitoneally. Core body temperature was maintained at 37.0 ± 0.1 °C using a homeothermic heating pad system (FHC, Inc., Bowdoin, ME). Linear acceleration pulses, 2 ms duration, were generated and controlled with National Instruments processors and presented to the cranium via a non-invasive spring clip that encircled the head and secured it to a voltage-controlled mechanical shaker. Stimuli were presented along the naso-occipital axis using two stimulus polarities, normal (upward pulse) and inverted (downward pulse). Stimuli were presented at a rate of 17 pulses/sec with amplitudes ranging from +6 dB to −18 dB re: 1.0 g/ms (where 1 g = 9.8 m/s^2^) adjusted in 3 dB steps. Electrodes were placed subcutaneously at the nuchal crest to serve as the noninverting electrode, and posterior to the left pinna and at the hip for inverting and ground electrodes, respectively. Traditional signal averaging was used to resolve responses in electrophysiological recordings. Ongoing electroencephalographic activity was amplified (equivalent to 200,000X), filtered (300 to 3000 Hz) and digitized (24 kHz sampling rate). Primary responses (n = 512) were averaged for each VsEP response waveform. All responses were replicated. VsEP intensity series was collected beginning at the maximum stimulus intensity (+6 dB re: 1.0 g/ms) with and without acoustic masking, then descending in 3 dB steps to −18 dB re: 1.0 g/ms. A broad band forward masker (50 to 50,000 Hz, 94 dB SPL) was presented during VsEP measurements to verify absence of cochlear responses^49^. VsEP thresholds were defined as the intensity midway between the minimum stimulus intensity that produced a discernable response and the maximum intensity where no response was detectable. Thresholds were compared between heterozygote and wildtype controls using two factor analysis of variance with age and genotype as the factors (SPSS Version 23.0).

### Auditory brainstem responses (ABRs)

For ABR testing, mice were anesthetized with pharmaceutical grade ketamine (100–150 mg/kg) and xylazine (10–16 mg/kg) solution by intraperitoneal injection. Recording electrodes were attached posterior to the right and left pinna, a noninverting electrode was attached at the vertex of the skull, and a ground electrode was attached to the hip. Hearing thresholds were determined at 8, 16, 24 and 32 kHz using an ABR recording system (Tucker-Davis Technologies). 500 sweeps of 5 ms tone bursts were introduced beginning at 90 dB peSPL (peak equivalent Sound Pressure Level) and proceeding in 5 dB decrements down to 0 dB peSPL. All recordings were performed in a soundproof box. Statistical significance between *Gfi1*^*Cre*^ heterozygote and wild-type groups was assessed by two-way ANOVA, followed by Tukey’s test to account for multiple comparisons.

### RNA sequencing and analysis

RNA was extracted from cochlear ducts of eight day old (P8) *Gfi1*^*Cre/*+^ and *Gfi1*^+/+^ mice in independent biological triplicate samples for each genotype using the Direct-zol RNA Mini-prep kit (Zymo Research, Irvine, CA). Each sequenced *Gfi1*^+/+^ sample consisted of pooled RNA from two cochleae (1 mouse), and each *Gfi1*^*Cre/*+^ sample consisted of pooled RNA from six cochleae (3 mice). RNA-seq libraries were prepared from a minimum of 75ng of RNA with the TruSeq RNA Sample Prep kit (Illumina) per manufacturer’s protocol, and samples were sequenced on an Illumina HiSeq 2500 with a paired-end 150 base configuration (between 38 and 50 million reads were obtained for each sample, see Supplementary Table 2). Sequenced reads were aligned to the mouse genome (mm10) using Tophat^50^. Only uniquely mapped reads were used in subsequent analyses. Gene expression levels were calculated using HTseq-count^51^ and normalized using quantile normalization. Only genes covered by at least 40 reads were considered as expressed in our dataset (14,866 genes). Differential expression analysis was done using DEseq^52^. RNA-seq data was submitted to the Gene Expression Omnibus database (accession ID GSE85519). Gene expression data from mouse macrophages were taken from GEO RNA-seq datasets GSE65530 and GSE77885^33^,^34^. Only unstimulated samples were analyzed. Genes covered by at least 40 reads were called as expressed genes in these datasets. Quantile normalization was applied to allow comparison of expression levels between different datasets. The definition of the set of genes whose expression is enriched in macrophages was based on the comparison of expression between macrophages and sensory epithelial, neuronal, mesenchymal and endothelial cells in the inner ear taken from Hertzano *et al*.^21^. 360 genes showed at least 3-fold elevated expression in macrophages compared to all other cell types.

### Quantitative RT-PCR

Total RNA was extracted from whole cochlear tissues from newborn (P0-P1) *Gfi1*^*Cre/*+^ and *Gfi1*^+/+^ mice using the Direct-zol RNA MiniPrep Kit (Zymo Research), and RNA was reverse-transcribed using the Maxima First Strand cDNA Synthesis Kit (Thermo Fisher Scientific). RT-qPCR was performed using Maxima SYBR Green/ROX qPCR Master Mix (Thermo Fisher Scientific) and primers designed for shared and unique exons between the wild-type and *Gfi1*^*Cre*^ knock-in alleles. These primers are as follows: exons 2 to 3 (Ex2–3 forward 5′-CCG AGT TCG AGG ACT TTT GGA-3′, Ex2-3 reverse 5′-AGC GGC ACA GTG ACT TCT C-3′), exons 5 to 6 (Ex5-6 forward 5′-AAT GCA GCA AGG TGT TCT-3′, Ex5-6 reverse 5′-CTT ACA GTC AAA GCT GCG T-3′), and exons 6 to 7 (Ex6-7 forward 5′-CAG GTG AGA AGC CCC ACA AA-3′, Ex6-7 reverse 5′-GAA GCC TGT GTG CTT TCT GC-3′). Transcript levels were normalized to *Myo7a* expression to account for possible differences in dissection and HC number as each biological replicate consisted of two cochleae only (myo7a forward 5′-GAA TCC ACG GTA CAG GGC AA-3′, myo7a reverse 5′-AGT AAA TCT CGT CCC GCA GC-3′). RT-qPCR was performed in 4 biological replicates, and statistical significance was assessed by Welch’s t-test.

## Additional Information

**How to cite this article:** Matern, M. *et al*. *Gfi1^Cre^* mice have early onset progressive hearing loss and induce recombination in numerous inner ear non-hair cells. *Sci. Rep.* **7**, 42079; doi: 10.1038/srep42079 (2017).

**Publisher's note:** Springer Nature remains neutral with regard to jurisdictional claims in published maps and institutional affiliations.

## Supplementary Material

Supplementary Information

## Figures and Tables

**Table 1:**
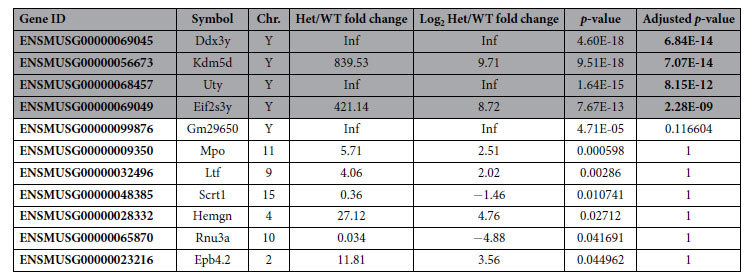
RNA-seq analysis of P8 *Gfi1*^*Cre/*+^ and *Gfi1*^+/+^ cochlear RNA.

Out of 14,866 genes found to be expressed in cochleae of the *Gfi1*^*Cre/*+^ (Het) and *Gfi1*^+/+^ (WT) mice, only 11 passed filtering for differential expression with standard criteria (Log_2_ fold change >1 or <−1, *p*-value < 0.05). Furthermore, only 4 genes (shaded in grey) could be considered significantly changed after taking into account multiple testing (false discovery rate, FDR = 0.05), all which are encoded on chromosome (Chr.) Y. Inf indicates infinity, or genes that were highly expressed in *Gfi1*^*Cre/*+^ samples and not detected in *Gfi1*^+/+^ samples.
