# Supplementary material for: Gfi1Cre mice have early onset progressive hearing loss and induce recombination in numerous inner ear non-hair cells
Source: Sci Rep. 2017 Feb 9;7:42079. doi: 10.1038/srep42079 (PMC5299610; doi:10.1038/srep42079)
Supplement: Supplementary Information [file srep42079-s1.pdf]

Supplementary figures and tables for:

Gfi1-Cre mice have early onset progressive hearing loss and induce recombination in numerous inner ear non-hair cells

Maggie Matern[1], Sarath Vijayakumar[2], Zachary Margulies[1], Beatrice Milon[1], Yang Song[3], Ran Elkon[4], Xiaoyu Zhang[1], Sherri M. Jones[2], and Ronna Hertzano*[1,3,5]

<u>Supplementary Figure 1</u> One representative FACS analysis of cochlear single cell suspensions from four day old *Gfi1*$^{Cre/+}$*;ROSA26*$^{CAG\text{-}tdTomato}$ mice, analyzing expression of CD326 and CD45 in the tdTomato positive cell population. On average, 95.2% of CD45 positive cells were also positive for tdTomato (P1, immune cells).

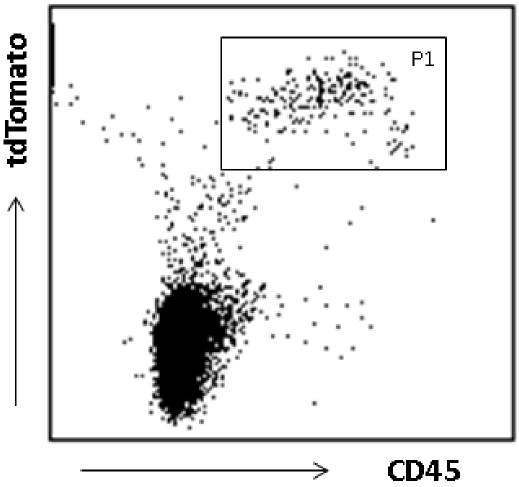

<u>Supplementary Figure 2</u> Quantitative comparative analysis of *Gfi1* transcript in *Gfi1*$^{+/+}$ and *Gfi1*$^{Cre/+}$ cochlea. RT-qPCR data showing relative expression levels of shared and unique exons between the wild-type and *Gfi1*^{Cre} knock-in alleles in the cochlea of newborn *Gfi1*$^{+/+}$ and *Gfi1*$^{Cre/+}$ littermates. A statistically significant ~30-40% decrease of *Gfi1* expression within regions substituted by Cre recombinase can be observed in *Gfi1*$^{Cre/+}$ mice cochlea compared to wild-type: exons 2 to 3 (Ex2-3: fold change = 0.675, *p*-value = 0.022), and exons 5 to 6 (Ex5-6: fold change = 0.621, *p*-value = 0.005). No significant change in abundance was observed for a region shared between the two genotypes (Ex6-7: fold change = 1.099, *p*-value = 0.52). N=4. Error bars represent fold change values taking into account one standard deviation. Statistical significance was assessed using Welch's t-test.

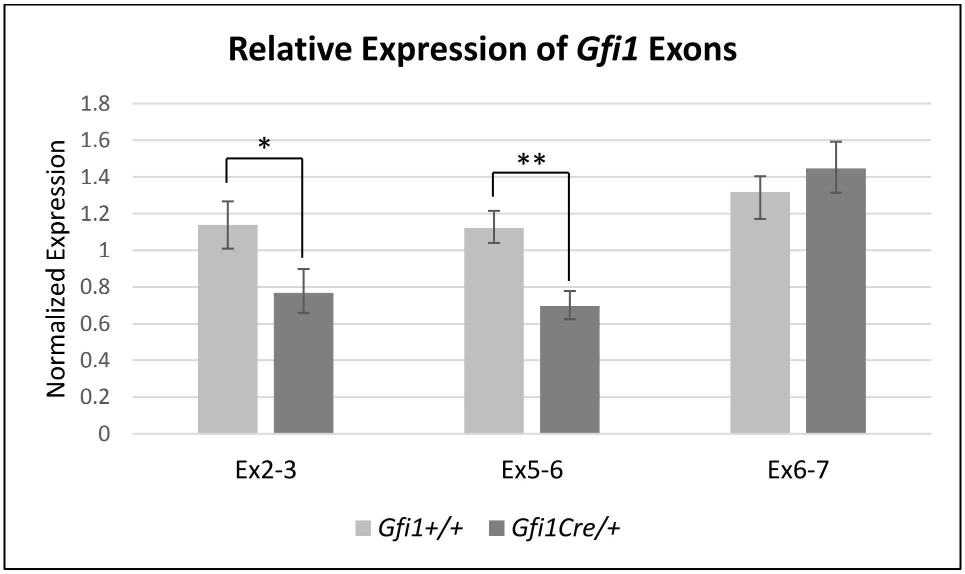

Supplementary Table 1 List of genes encoding 521 transcripts enriched in HCs according to both Elkon et al. and Cai et al.[1,32]

**Ensembl ID**	**Gene symbol**
ENSMUSG00000037971	1110032A03Rik
ENSMUSG00000046999	1110032F04Rik
ENSMUSG00000031847	1700030J22Rik
ENSMUSG00000062760	1810041L15Rik
ENSMUSG00000026090	2010300C02Rik
ENSMUSG00000071632	2510002D24Rik
ENSMUSG00000028300	3110043O21Rik
ENSMUSG00000046567	4930430F08Rik
ENSMUSG00000050549	5730508B09Rik
ENSMUSG00000079499	6530402F18Rik
ENSMUSG00000045838	A430105I19Rik
ENSMUSG00000023088	Abcc1
ENSMUSG00000055782	Abcd2
ENSMUSG00000026644	Acbd7
ENSMUSG00000028937	Acot7
ENSMUSG00000031278	Acsl4
ENSMUSG00000027605	Acss2
ENSMUSG00000056367	Actr3b
ENSMUSG00000020926	Adam11
ENSMUSG00000022376	Adcy8
ENSMUSG00000031785	Adgrg1
ENSMUSG00000069170	Adgrv1
ENSMUSG00000046312	AI464131
ENSMUSG00000040407	Akap9
ENSMUSG00000005686	Ampd3
ENSMUSG00000024774	Ankrd22
ENSMUSG00000054708	Ankrd24
ENSMUSG00000058589	Anks1b
ENSMUSG00000074968	Ano3
ENSMUSG00000029994	Anxa4
ENSMUSG00000062444	Ap3b2
ENSMUSG00000024897	Apba1
ENSMUSG00000006651	Aplp1
ENSMUSG00000021662	Arhgef28
ENSMUSG00000023017	Asic1
ENSMUSG00000026663	Atf6

ENSMUSG00000031441	Atp11a
ENSMUSG00000031376	Atp2b3
ENSMUSG00000019302	Atp6v0a1
ENSMUSG00000021983	Atp8a2
ENSMUSG00000018126	Baiap2l2
ENSMUSG00000026805	Barhl1
ENSMUSG00000006464	Bbs1
ENSMUSG00000063145	Bbs5
ENSMUSG00000034773	BC030867
ENSMUSG00000000861	Bcl11a
ENSMUSG00000048482	Bdnf
ENSMUSG00000092060	Bend4
ENSMUSG00000027358	Bmp2
ENSMUSG00000035390	Brsk1
ENSMUSG00000053046	Brsk2
ENSMUSG00000032589	Bsn
ENSMUSG00000040565	Btaf1
ENSMUSG00000020042	Btbd11
ENSMUSG00000000202	Btbd17
ENSMUSG00000032120	C2cd2l
ENSMUSG00000050390	C77080
ENSMUSG00000057914	Cacnb2
ENSMUSG00000019146	Cacng2
ENSMUSG00000028222	Calb1
ENSMUSG00000003657	Calb2
ENSMUSG00000030669	Calca
ENSMUSG00000001175	Calm1
ENSMUSG00000033170	Card10
ENSMUSG00000010755	Cars
ENSMUSG00000028977	Casz1
ENSMUSG00000026578	Ccdc181
ENSMUSG00000027676	Ccdc39
ENSMUSG00000039963	Ccdc40
ENSMUSG00000003354	Ccdc65
ENSMUSG00000038903	Ccdc68
ENSMUSG00000021182	Ccdc88c
ENSMUSG00000050677	Ccdc96
ENSMUSG00000033904	Ccp110
ENSMUSG00000058690	Ccser2

ENSMUSG00000028865	Cd164l2
ENSMUSG00000026490	Cdc42bpa
ENSMUSG00000024304	Cdh2
ENSMUSG00000012819	Cdh23
ENSMUSG00000026023	Cdk15
ENSMUSG00000020015	Cdk17
ENSMUSG00000039298	Cdk5rap2
ENSMUSG00000020990	Cdkl1
ENSMUSG00000033966	Cdkl4
ENSMUSG00000031758	Cdyl2
ENSMUSG00000023473	Celsr3
ENSMUSG00000035790	Cep19
ENSMUSG00000035891	Cerk
ENSMUSG00000031347	Cetn2
ENSMUSG00000028294	Cfap206
ENSMUSG00000040473	Cfap69
ENSMUSG00000041235	Chd7
ENSMUSG00000021194	Chga
ENSMUSG00000027350	Chgb
ENSMUSG00000027107	Chrna1
ENSMUSG00000066279	Chrna10
ENSMUSG00000029205	Chrna9
ENSMUSG00000027950	Chrnb2
ENSMUSG00000035200	Chrnb4
ENSMUSG00000037493	Cib2
ENSMUSG00000000308	Ckmt1
ENSMUSG00000047109	Cldn14
ENSMUSG00000033633	Clec18a
ENSMUSG00000032245	Cln6
ENSMUSG00000047547	Cltb
ENSMUSG00000025658	Cnksr2
ENSMUSG00000018661	Cog1
ENSMUSG00000026798	Coq4
ENSMUSG00000037852	Cpe
ENSMUSG00000025586	Cpeb1
ENSMUSG00000039652	Cpeb3
ENSMUSG00000033615	Cplx1
ENSMUSG00000029121	Crmp1
ENSMUSG00000022240	Ctnnd2

ENSMUSG00000026726	Cubn
ENSMUSG00000021508	Cxcl14
ENSMUSG00000019590	Cyb561
ENSMUSG00000026456	Cyb5r1
ENSMUSG00000031865	Dctn1
ENSMUSG00000027173	Depdc7
ENSMUSG00000075267	Dfnb59
ENSMUSG00000034731	Dgkh
ENSMUSG00000040035	Disp2
ENSMUSG00000052572	Dlg2
ENSMUSG00000047428	Dlk2
ENSMUSG00000014773	Dll1
ENSMUSG00000027314	Dll4
ENSMUSG00000041268	Dmxl2
ENSMUSG00000020973	Dnaaf2
ENSMUSG00000018581	Dnah11
ENSMUSG00000033826	Dnah8
ENSMUSG00000034706	Dnaic2
ENSMUSG00000030708	Dnajb13
ENSMUSG00000036766	Dner
ENSMUSG00000040265	Dnm3
ENSMUSG00000039716	Dock3
ENSMUSG00000057147	Dph6
ENSMUSG00000029005	Draxin
ENSMUSG00000032087	Dscaml1
ENSMUSG00000024302	Dtna
ENSMUSG00000018648	Dusp14
ENSMUSG00000047193	Dync2h1
ENSMUSG00000034467	Dynlrb2
ENSMUSG00000069085	Dytn
ENSMUSG00000037259	Dzank1
ENSMUSG00000045071	E130308A19Rik
ENSMUSG00000041809	Efhc1
ENSMUSG00000015002	Efr3a
ENSMUSG00000048988	Elfn1
ENSMUSG00000043460	Elfn2
ENSMUSG00000041986	Elmod1
ENSMUSG00000022012	Enox1
ENSMUSG00000025504	Eps8l2

ENSMUSG00000054051	Ercc6
ENSMUSG00000028943	Espn
ENSMUSG00000049515	Espnl
ENSMUSG00000026610	Esrrg
ENSMUSG00000053799	Exoc6
ENSMUSG00000029461	Fam168a
ENSMUSG00000055865	Fam19a3
ENSMUSG00000048458	Fam212b
ENSMUSG00000070476	Fam217b
ENSMUSG00000058966	Fam57b
ENSMUSG00000060568	Fam78b
ENSMUSG00000025738	Fbxl16
ENSMUSG00000034532	Fbxo16
ENSMUSG00000073633	Fbxo36
ENSMUSG00000001366	Fbxo9
ENSMUSG00000070524	Fcrlb
ENSMUSG00000032118	Fez1
ENSMUSG00000060579	Fhit
ENSMUSG00000034295	Fhod3
ENSMUSG00000025175	Fn3k
ENSMUSG00000038368	Focad
ENSMUSG00000034227	Foxj1
ENSMUSG00000070733	Fryl
ENSMUSG00000054752	Fsd1l
ENSMUSG00000036264	Fstl4
ENSMUSG00000026787	Gad2
ENSMUSG00000060988	Galnt13
ENSMUSG00000024064	Galnt14
ENSMUSG00000031451	Gas6
ENSMUSG00000021360	Gcnt2
ENSMUSG00000025777	Gdap1
ENSMUSG00000029275	Gfi1
ENSMUSG00000031748	Gnao1
ENSMUSG00000034707	Gns
ENSMUSG00000041762	Gpr155
ENSMUSG00000046961	Gpr156
ENSMUSG00000044317	Gpr4
ENSMUSG00000010825	Grid2ip
ENSMUSG00000031450	Grk1

ENSMUSG00000024517	Grp
ENSMUSG00000024580	Grpel2
ENSMUSG00000038515	Grtp1
ENSMUSG00000068082	Grxcr1
ENSMUSG00000073574	Grxcr2
ENSMUSG00000004035	Gstm7
ENSMUSG00000042535	Gtpbp1
ENSMUSG00000033629	Hacd3
ENSMUSG00000019853	Hebp2
ENSMUSG00000021301	Hecw1
ENSMUSG00000075254	Heg1
ENSMUSG00000067071	Hes6
ENSMUSG00000018102	Hist1h2bc
ENSMUSG00000028572	Hook1
ENSMUSG00000046607	Hrk
ENSMUSG00000070407	Hs3st3b1
ENSMUSG00000026675	Hsd17b7
ENSMUSG00000021270	Hsp90aa1
ENSMUSG00000025757	Hspa4l
ENSMUSG00000059854	Hydin
ENSMUSG00000062995	Ica1
ENSMUSG00000026018	Ica1l
ENSMUSG00000024169	Ift140
ENSMUSG00000038564	Ift172
ENSMUSG00000024474	Ik
ENSMUSG00000034336	Ina
ENSMUSG00000041324	Inhba
ENSMUSG00000048782	Insc
ENSMUSG00000060798	Intu
ENSMUSG00000035578	Iqcg
ENSMUSG00000042644	Itpr3
ENSMUSG00000002799	Jag2
ENSMUSG00000042861	Kcna10
ENSMUSG00000042604	Kcna4
ENSMUSG00000050556	Kcnb1
ENSMUSG00000038319	Kcnh2
ENSMUSG00000001901	Kcnh6
ENSMUSG00000059742	Kcnh7
ENSMUSG00000035580	Kcnh8

ENSMUSG00000054477	Kcnn2
ENSMUSG00000028631	Kcnq4
ENSMUSG00000024201	Kdm4b
ENSMUSG00000010021	Kif19a
ENSMUSG00000041642	Kif21b
ENSMUSG00000028438	Kif24
ENSMUSG00000074657	Kif5a
ENSMUSG00000026764	Kif5c
ENSMUSG00000032489	Kif9
ENSMUSG00000091680	Klhdc7b
ENSMUSG00000073774	Kncn
ENSMUSG00000066129	Kndc1
ENSMUSG00000035849	Krt222
ENSMUSG00000032370	Lactb
ENSMUSG00000028581	Laptm5
ENSMUSG00000060594	Layn
ENSMUSG00000042873	Lhfpl4
ENSMUSG00000062252	Lhfpl5
ENSMUSG00000026934	Lhx3
ENSMUSG00000023022	Lima1
ENSMUSG00000044626	Liph
ENSMUSG00000039704	Lmbrd2
ENSMUSG00000033060	Lmo7
ENSMUSG00000048096	Lmod1
ENSMUSG00000044086	Lmod3
ENSMUSG00000016520	Lnx2
ENSMUSG00000032818	Loxhd1
ENSMUSG00000056215	Lrguk
ENSMUSG00000019796	Lrp11
ENSMUSG00000028613	Lrp8
ENSMUSG00000015980	Lrrc27
ENSMUSG00000056598	Lrrc48
ENSMUSG00000071073	Lrrc73
ENSMUSG00000055003	Lrtm2
ENSMUSG00000022577	Ly6h
ENSMUSG00000033900	Map9
ENSMUSG00000022619	Mapk8ip2
ENSMUSG00000051098	Mblac2
ENSMUSG00000028910	Mecr

ENSMUSG00000036466	Megf11
ENSMUSG00000018169	Mfng
ENSMUSG00000041945	Mfsd9
ENSMUSG00000043857	Mgat5b
ENSMUSG00000039478	Micu3
ENSMUSG00000068011	Mkrn2os
ENSMUSG00000068036	Mllt4
ENSMUSG00000027612	Mmp24
ENSMUSG00000032221	Mns1
ENSMUSG00000039395	Mreg
ENSMUSG00000051236	Msrb3
ENSMUSG00000005667	Mthfd2
ENSMUSG00000045934	Mtmr11
ENSMUSG00000039431	Mtmr7
ENSMUSG00000039110	Mycbpap
ENSMUSG00000028654	Mycl
ENSMUSG00000039057	Myo16
ENSMUSG00000000631	Myo18a
ENSMUSG00000066952	Myo1h
ENSMUSG00000025716	Myo3a
ENSMUSG00000042064	Myo3b
ENSMUSG00000033577	Myo6
ENSMUSG00000030761	Myo7a
ENSMUSG00000024471	Myot
ENSMUSG00000047490	na
ENSMUSG00000027438	Napb
ENSMUSG00000020576	Nbas
ENSMUSG00000050323	Ndufaf6
ENSMUSG00000032012	Nectin1
ENSMUSG00000022055	Nefl
ENSMUSG00000022054	Nefm
ENSMUSG00000035032	Nek11
ENSMUSG00000034413	Neurl1b
ENSMUSG00000026442	Nfasc
ENSMUSG00000051251	Nhlh1
ENSMUSG00000021068	Nin
ENSMUSG00000035984	Nme5
ENSMUSG00000032456	Nmnat3
ENSMUSG00000020598	Nrcam

ENSMUSG00000048978	Nrsn1
ENSMUSG00000066392	Nrxn3
ENSMUSG00000035513	Ntng2
ENSMUSG00000030091	Nup210
ENSMUSG00000048148	Nwd1
ENSMUSG00000061462	Obscn
ENSMUSG00000027848	Olfml3
ENSMUSG00000039747	Orai2
ENSMUSG00000038150	Ormdl3
ENSMUSG00000029822	Osbpl3
ENSMUSG00000042616	Oscp1
ENSMUSG00000062372	Otof
ENSMUSG00000041161	Otud3
ENSMUSG00000036990	Otud4
ENSMUSG00000027071	P2rx3
ENSMUSG00000037196	Pacrg
ENSMUSG00000040276	Pacsin1
ENSMUSG00000031284	Pak3
ENSMUSG00000064225	Paqr9
ENSMUSG00000026496	Parp1
ENSMUSG00000001120	Pcbp3
ENSMUSG00000025050	Pcgf6
ENSMUSG00000061601	Pclo
ENSMUSG00000031592	Pcm1
ENSMUSG00000034501	Pcnx4
ENSMUSG00000038370	Pcp4l1
ENSMUSG00000041837	Pdcd7
ENSMUSG00000074818	Pdzd7
ENSMUSG00000035357	Pdzrn3
ENSMUSG00000021196	Pfkp
ENSMUSG00000020475	Pgam2
ENSMUSG00000039987	Phtf2
ENSMUSG00000041482	Piezo2
ENSMUSG00000026737	Pip4k2a
ENSMUSG00000017781	Pitpna
ENSMUSG00000038725	Pkhd1l1
ENSMUSG00000026785	Pkn3
ENSMUSG00000060675	Pla2g16
ENSMUSG00000024998	Plce1

ENSMUSG00000029055	Plch2
ENSMUSG00000051344	Plekhm3
ENSMUSG00000046204	Pnma2
ENSMUSG00000031536	Polb
ENSMUSG00000024497	Pou4f3
ENSMUSG00000033871	Ppargc1b
ENSMUSG00000078451	Ppil6
ENSMUSG00000046442	Ppm1e
ENSMUSG00000034613	Ppm1h
ENSMUSG00000028518	Prkaa2
ENSMUSG00000021948	Prkcd
ENSMUSG00000035725	Prkx
ENSMUSG00000039449	Prpf18
ENSMUSG00000023978	Prph2
ENSMUSG00000047040	Prr15l
ENSMUSG00000045009	Prrt3
ENSMUSG00000024640	Psat1
ENSMUSG00000043017	Ptgir
ENSMUSG00000038764	Ptpn3
ENSMUSG00000056553	Ptprn2
ENSMUSG00000035916	Ptprq
ENSMUSG00000005716	Pvalb
ENSMUSG00000031488	Rab11fip1
ENSMUSG00000020175	Rab36
ENSMUSG00000003411	Rab3b
ENSMUSG00000064181	Rab3ip
ENSMUSG00000032549	Rab6b
ENSMUSG00000061311	Rag1
ENSMUSG00000034472	Rasd2
ENSMUSG00000035275	Raver2
ENSMUSG00000038132	Rbm24
ENSMUSG00000021123	Rdh12
ENSMUSG00000052852	Reep1
ENSMUSG00000005873	Reep5
ENSMUSG00000024186	Rgs11
ENSMUSG00000029101	Rgs12
ENSMUSG00000048330	Ric3
ENSMUSG00000029420	Rimbp2
ENSMUSG00000037386	Rims2

ENSMUSG00000032890	Rims3
ENSMUSG00000031438	Rnf128
ENSMUSG00000047747	Rnf150
ENSMUSG00000052949	Rnf157
ENSMUSG00000044164	Rnf182
ENSMUSG00000029130	Rnf32
ENSMUSG00000029608	Rph3a
ENSMUSG00000075334	Rprm
ENSMUSG00000024033	Rsph1
ENSMUSG00000029291	Rufy3
ENSMUSG00000006575	Rundc3a
ENSMUSG00000021313	Ryr2
ENSMUSG00000042312	S100a13
ENSMUSG00000038570	Saxo2
ENSMUSG00000038936	Sccpdh
ENSMUSG00000023236	Scg5
ENSMUSG00000075702	Selm
ENSMUSG00000063531	Sema3e
ENSMUSG00000052133	Sema5b
ENSMUSG00000026315	Serpinb8
ENSMUSG00000032908	Sgpp2
ENSMUSG00000037833	Sh2d4b
ENSMUSG00000037541	Shank2
ENSMUSG00000035378	Shq1
ENSMUSG00000045180	Shroom2
ENSMUSG00000017740	Slc12a5
ENSMUSG00000019935	Slc17a8
ENSMUSG00000036330	Slc18a1
ENSMUSG00000029015	Slc26a5
ENSMUSG00000072572	Slc39a2
ENSMUSG00000027075	Slc43a1
ENSMUSG00000030307	Slc6a11
ENSMUSG00000027894	Slc6a17
ENSMUSG00000069072	Slc7a14
ENSMUSG00000030376	Slc8a2
ENSMUSG00000026062	Slc9a2
ENSMUSG00000031129	Slc9a9
ENSMUSG00000041476	Smpx
ENSMUSG00000027273	Snap25

ENSMUSG00000023064	Sncg
ENSMUSG00000027457	Snph
ENSMUSG00000025006	Sorbs1
ENSMUSG00000001948	Spa17
ENSMUSG00000072663	Spef2
ENSMUSG00000041974	Spidr
ENSMUSG00000024533	Spire1
ENSMUSG00000058297	Spock2
ENSMUSG00000021061	Sptb
ENSMUSG00000021594	Srd5a1
ENSMUSG00000063919	Srrm4
ENSMUSG00000025789	St8sia2
ENSMUSG00000056812	St8sia3
ENSMUSG00000030688	Stard10
ENSMUSG00000020303	Stc2
ENSMUSG00000033498	Strc
ENSMUSG00000018865	Sult4a1
ENSMUSG00000038486	Sv2a
ENSMUSG00000059602	Syn3
ENSMUSG00000019737	Syne4
ENSMUSG00000034940	Synrg
ENSMUSG00000031144	Syp
ENSMUSG00000027220	Syt13
ENSMUSG00000016200	Syt14
ENSMUSG00000026452	Syt2
ENSMUSG00000024261	Syt4
ENSMUSG00000051397	Tacstd2
ENSMUSG00000052302	Tbc1d30
ENSMUSG00000091002	Tcerg1l
ENSMUSG00000020799	Tekt1
ENSMUSG00000029359	Tesc
ENSMUSG00000042581	Thsd7b
ENSMUSG00000012296	Tjap1
ENSMUSG00000024749	Tmc1
ENSMUSG00000042066	Tmcc2
ENSMUSG00000054434	Tmem120b
ENSMUSG00000024349	Tmem173
ENSMUSG00000057716	Tmem178b
ENSMUSG00000054013	Tmem179

ENSMUSG00000036687	Tmem184a
ENSMUSG00000026627	Tmem206
ENSMUSG00000024667	Tmem216
ENSMUSG00000048022	Tmem229a
ENSMUSG00000031951	Tmem231
ENSMUSG00000022856	Tmem41a
ENSMUSG00000030059	Tmf1
ENSMUSG00000049555	Tmie
ENSMUSG00000033177	Tmprss7
ENSMUSG00000028364	Tnc
ENSMUSG00000018322	Tomm34
ENSMUSG00000033949	Trim36
ENSMUSG00000041000	Trim62
ENSMUSG00000033088	Triobp
ENSMUSG00000056596	Trnp1
ENSMUSG00000028251	Tstd3
ENSMUSG00000032514	Ttc21a
ENSMUSG00000051036	Ttc24
ENSMUSG00000038172	Ttc39b
ENSMUSG00000030276	Ttll3
ENSMUSG00000036745	Ttll7
ENSMUSG00000005968	Tuft1
ENSMUSG00000010054	Tusc2
ENSMUSG00000023277	Twf2
ENSMUSG00000034485	Uaca
ENSMUSG00000036352	Ubac1
ENSMUSG00000052981	Ube2ql1
ENSMUSG00000043621	Ubxn10
ENSMUSG00000032854	Ugt8a
ENSMUSG00000034799	Unc13a
ENSMUSG00000021198	Unc79
ENSMUSG00000055567	Unc80
ENSMUSG00000043241	Upf2
ENSMUSG00000026609	Ush2a
ENSMUSG00000026854	Usp20
ENSMUSG00000054814	Usp46
ENSMUSG00000047712	Ust
ENSMUSG00000035284	Vps13c
ENSMUSG00000046613	Vwa5b2

ENSMUSG00000050830	Vwc2
ENSMUSG00000019831	Wasf1
ENSMUSG00000028391	Wdr31
ENSMUSG00000035126	Wdr78
ENSMUSG00000029029	Wrap73
ENSMUSG00000018849	Wwc1
ENSMUSG00000030657	Xylt1
ENSMUSG00000060862	Zbtb40
ENSMUSG00000030471	Zdhhc13
ENSMUSG00000025157	Zdhhc16
ENSMUSG00000001065	Zfp276
ENSMUSG00000037855	Zfp365
ENSMUSG00000000552	Zfp385a
ENSMUSG00000039081	Zfp503
ENSMUSG00000054893	Zfp667
ENSMUSG00000039238	Zfp750
ENSMUSG00000037492	Zmat4

Supplementary Table 2 Summary data for *Gfi1*$^{Cre/+}$ (HET1-3) and *Gfi1*$^{+/+}$ (WT1-3) RNA-seq samples.

Sample ID	Total reads	Total mapped reads	% mapped reads	% properly paired	Uniquely mapped reads	% exonic	% intronic	% intergenic	Total features	Features with coverage	Average RPKM
HET1	49458954	44653988	90.28	95.36	42632568	93.02	4.54	2.44	43620	25366	6.365
HET2	47591802	43262912	90.9	93.54	41438699	93.07	4.55	2.38	43620	25218	6.042
HET3	45775884	41202266	90.01	95.29	39367365	93.45	4.12	2.43	43620	25126	6.312
WT1	38491984	33187310	86.22	94.52	31444234	91.33	4.79	3.88	43620	25675	8.193
WT2	40764300	36770716	90.2	95.61	35038788	93.08	4.46	2.46	43620	24908	6.736
WT3	42862376	38067548	88.81	95.33	36389984	91.86	5.14	2.99	43620	25205	6.458

Supplementary Figure 3 **A.** Expression of the set of 521 HC-enriched transcripts (used in the analysis shown in Figure 6C) is significantly elevated in HCs also when compared to macrophages (Mph). To address a possible concern that the downregulation of the HC-enriched transcripts in *Gfi1*$^{Cre/+}$ cochleae could arise from changes in Mph gene expression, we further determined the relative expression levels of these 521 transcripts in two Mph datasets. For each gene that was expressed in both the HC dataset (based on intersection of Elkon et al., Cai et al.[1,32]) and the Mph datasets (obtained from Avraham et al., Langlais et al.[33,34]), HC to Mph fold-change of expression level was calculated. This resulted in a set of fold-change values for 7,219 and 9,078 genes expressed both in HCs and Mph. Of the 521 HC-enriched transcripts defined in Fig 6C, only 135 and 254 were expressed in the two Mph datasets, respectively. This analysis compares the distribution of fold-change values for these remaining HC-enriched transcripts vs. all other overlapping genes (*p*-values calculated using Wilcoxon's test.). Thus, the majority of the HC-enriched transcripts were not detected at all in Mph, and those that were detected (135 and 254) show significantly higher expression in HCs (*p*-values = 1.22E-20 and 2.71E-55 respectively). **B.** Based on the macrophage expression datasets, a set of 360 macrophage-enriched transcripts was defined. The expression of this set of genes is not decreased in the *Gfi1*$^{Cre/+}$ mice cochlea.

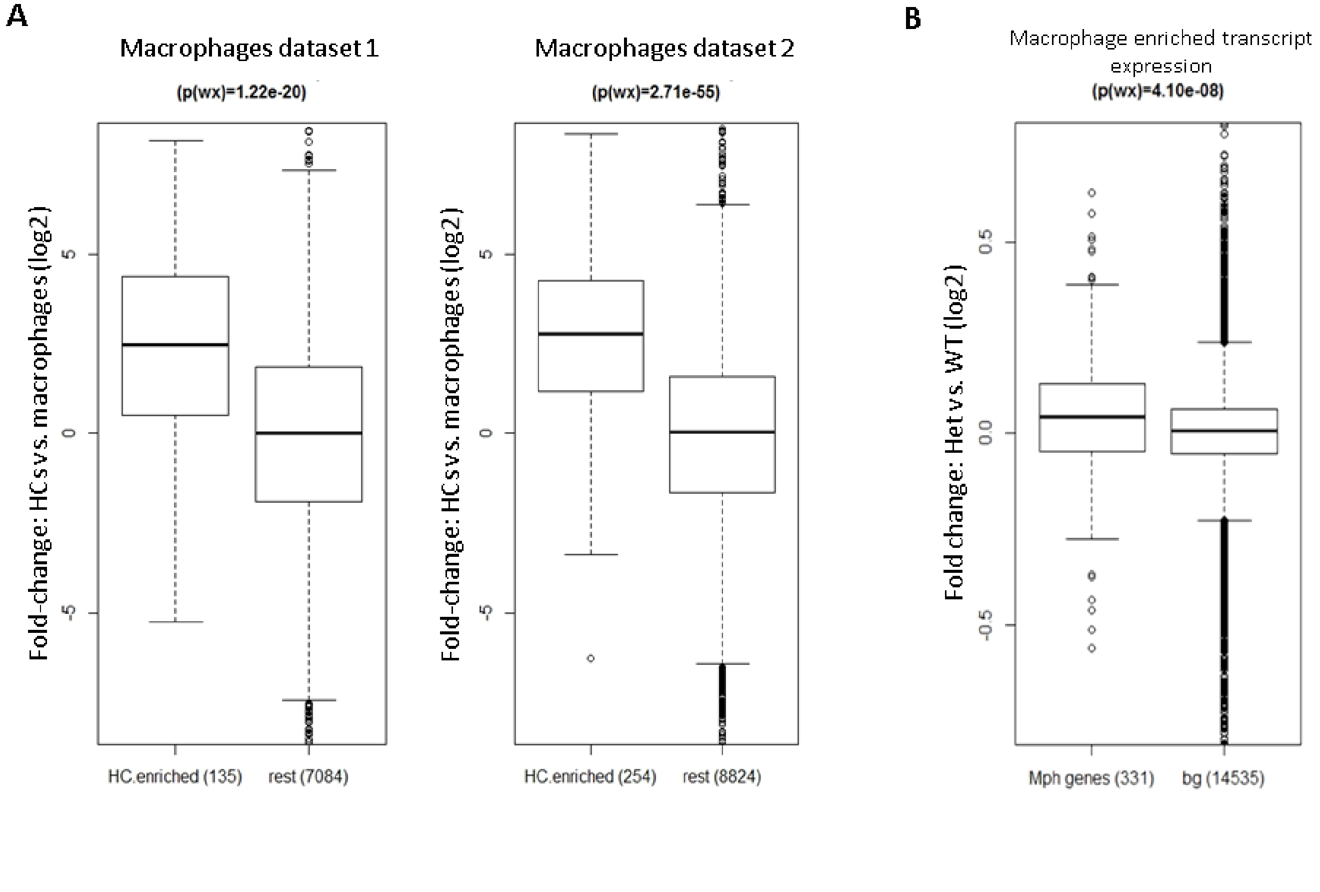

Supplementary Table 3 List of genes encoding 360 transcripts enriched in macrophages according to both Avraham et al., 2016 and Langlais et al., 2016[33,34].

Ensembl ID	Gene symbol
ENSMUSG00000069892	9930111J21Rik2
ENSMUSG00000052760	A630001G21Rik
ENSMUSG00000023088	Abcc1
ENSMUSG00000020865	Abcc3
ENSMUSG00000044573	Acp1
ENSMUSG00000062825	Actg1
ENSMUSG00000025473	Adam8
ENSMUSG00000056413	Adap1
ENSMUSG00000039041	Adrm1
ENSMUSG00000011148	Adssl1
ENSMUSG00000027597	Ahcy
ENSMUSG00000062753	AI413582
ENSMUSG00000087107	AI662270
ENSMUSG00000024397	Aif1
ENSMUSG00000037860	Aim2
ENSMUSG00000024885	Aldh3b1
ENSMUSG00000060063	Alox5ap
ENSMUSG00000030649	Anapc15
ENSMUSG00000040701	Ap1g2
ENSMUSG00000022841	Ap2m1
ENSMUSG00000026786	Apbb1ip
ENSMUSG00000040613	Apobec1
ENSMUSG00000027255	Arfgap2
ENSMUSG00000048865	Arhgap30
ENSMUSG00000031389	Arhgap4
ENSMUSG00000030220	Arhgdib
ENSMUSG00000060216	Arrb2
ENSMUSG00000005470	Asf1b
ENSMUSG00000029752	Asns
ENSMUSG00000006057	Atp5g1
ENSMUSG00000038717	Atp5l
ENSMUSG00000033379	Atp6v0b
ENSMUSG00000022295	Atp6v1c1
ENSMUSG00000060131	Atp8b4
ENSMUSG00000054428	Atpif1
ENSMUSG00000060802	B2m
ENSMUSG00000059479	B3gnt8
ENSMUSG00000006731	B4galnt1

ENSMUSG00000004446	Bid
ENSMUSG00000061132	Blnk
ENSMUSG00000001999	Blvra
ENSMUSG00000040466	Blvrb
ENSMUSG00000021660	Btf3
ENSMUSG00000036887	C1qa
ENSMUSG00000036905	C1qb
ENSMUSG00000036896	C1qc
ENSMUSG00000040552	C3ar1
ENSMUSG00000049130	C5ar1
ENSMUSG00000056737	Capg
ENSMUSG00000070372	Capza1
ENSMUSG00000029836	Cbx3
ENSMUSG00000047810	Ccdc88b
ENSMUSG00000035385	Ccl2
ENSMUSG00000000982	Ccl3
ENSMUSG00000018930	Ccl4
ENSMUSG00000035373	Ccl7
ENSMUSG00000019122	Ccl9
ENSMUSG00000051439	Cd14
ENSMUSG00000021624	Cd180
ENSMUSG00000002944	Cd36
ENSMUSG00000030798	Cd37
ENSMUSG00000040747	Cd53
ENSMUSG00000025351	Cd63
ENSMUSG00000018774	Cd68
ENSMUSG00000024610	Cd74
ENSMUSG00000026437	Cdk18
ENSMUSG00000056501	Cebpb
ENSMUSG00000001128	Cfp
ENSMUSG00000090258	Churc1
ENSMUSG00000032436	Cmtm7
ENSMUSG00000030707	Coro1a
ENSMUSG00000028337	Coro2a
ENSMUSG00000091803	Cox16
ENSMUSG00000000088	Cox5a
ENSMUSG00000061518	Cox5b
ENSMUSG00000017778	Cox7c
ENSMUSG00000040713	Creg1
ENSMUSG00000006360	Crip1
ENSMUSG00000024621	Csf1r

ENSMUSG00000059326	Csf2ra
ENSMUSG00000071714	Csf2rb2
ENSMUSG00000005054	Cstb
ENSMUSG00000038642	Ctss
ENSMUSG00000016256	Ctsz
ENSMUSG00000052336	Cx3cr1
ENSMUSG00000018920	Cxcl16
ENSMUSG00000032872	Cyb5r4
ENSMUSG00000006519	Cyba
ENSMUSG00000015340	Cybb
ENSMUSG00000063694	Cycs
ENSMUSG00000018008	Cyth4
ENSMUSG00000026832	Cytip
ENSMUSG00000030347	D6Wsu163e
ENSMUSG00000001666	Ddt
ENSMUSG00000001674	Ddx18
ENSMUSG00000013973	Dedd
ENSMUSG00000017830	Dhx58
ENSMUSG00000019088	Dnase1l1
ENSMUSG00000003812	Dnase2a
ENSMUSG00000020143	Dock2
ENSMUSG00000052085	Dock8
ENSMUSG00000068335	Dok1
ENSMUSG00000022102	Dok2
ENSMUSG00000035711	Dok3
ENSMUSG00000053687	Dpep2
ENSMUSG00000020057	Dram1
ENSMUSG00000071644	Eef1g
ENSMUSG00000040659	Efhd2
ENSMUSG00000035150	Eif2s3x
ENSMUSG00000059796	Eif4a1
ENSMUSG00000021728	Emb
ENSMUSG00000040212	Emp3
ENSMUSG00000021996	Esd
ENSMUSG00000027533	Fabp5
ENSMUSG00000056069	Fam105a
ENSMUSG00000083012	Fam220a
ENSMUSG00000038274	Fau
ENSMUSG00000046865	Fbl
ENSMUSG00000058715	Fcer1g
ENSMUSG00000059498	Fcgr3

ENSMUSG00000059743	Fdps
ENSMUSG00000024742	Fen1
ENSMUSG00000024965	Fermt3
ENSMUSG00000037946	Fgd3
ENSMUSG00000055805	Fmnl1
ENSMUSG00000033386	Frrs1
ENSMUSG00000024661	Fth1
ENSMUSG00000050708	Ftl1
ENSMUSG00000009687	Fxyd5
ENSMUSG00000022148	Fyb
ENSMUSG00000057666	Gapdh
ENSMUSG00000058624	Gda
ENSMUSG00000031266	Gla
ENSMUSG00000021591	Glrx
ENSMUSG00000078862	Gm14326
ENSMUSG00000078866	Gm14420
ENSMUSG00000060791	Gmfg
ENSMUSG00000036246	Gmip
ENSMUSG00000006715	Gmnn
ENSMUSG00000027523	Gnas
ENSMUSG00000068523	Gng5
ENSMUSG00000052102	Gnpda1
ENSMUSG00000029816	Gpnmb
ENSMUSG00000021306	Gpr137b
ENSMUSG00000025156	Gps1
ENSMUSG00000034786	Gpsm3
ENSMUSG00000063856	Gpx1
ENSMUSG00000037894	H2afz
ENSMUSG00000061232	H2-K1
ENSMUSG00000067212	H2-T23
ENSMUSG00000060743	H3f3a
ENSMUSG00000027944	Hax1
ENSMUSG00000003283	Hck
ENSMUSG00000022831	Hcls1
ENSMUSG00000028800	Hdac1
ENSMUSG00000021665	Hexb
ENSMUSG00000025877	Hk3
ENSMUSG00000003038	Hmgn2
ENSMUSG00000035697	Hmha1
ENSMUSG00000005413	Hmox1
ENSMUSG00000026986	Hnmt

ENSMUSG00000029919	Hpgds
ENSMUSG00000015656	Hspa8
ENSMUSG00000027245	Hypk
ENSMUSG00000079017	Ifi27l2a
ENSMUSG00000035004	Igsf6
ENSMUSG00000042349	Ikbke
ENSMUSG00000018654	Ikzf1
ENSMUSG00000032089	Il10ra
ENSMUSG00000022969	Il10rb
ENSMUSG00000002897	Il17ra
ENSMUSG00000030745	Il21r
ENSMUSG00000031304	Il2rg
ENSMUSG00000027947	Il6ra
ENSMUSG00000003882	Il7r
ENSMUSG00000062867	Impdh2
ENSMUSG00000029771	Irf5
ENSMUSG00000041515	Irf8
ENSMUSG00000030830	Itgal
ENSMUSG00000030786	Itgam
ENSMUSG00000000290	Itgb2
ENSMUSG00000028931	Kcnab2
ENSMUSG00000054342	Kcnn4
ENSMUSG00000018362	Kpna2
ENSMUSG00000055541	Lair1
ENSMUSG00000091512	Lamtor3
ENSMUSG00000028581	Laptm5
ENSMUSG00000040751	Lat2
ENSMUSG00000002699	Lcp2
ENSMUSG00000063229	Ldha
ENSMUSG00000050335	Lgals3
ENSMUSG00000033880	Lgals3bp
ENSMUSG00000024781	Lipa
ENSMUSG00000016239	Lonrf3
ENSMUSG00000015568	Lpl
ENSMUSG00000030263	Lrmp
ENSMUSG00000018819	Lsp1
ENSMUSG00000069516	Lyz2
ENSMUSG00000039208	Metrnl
ENSMUSG00000034854	Mfsd12
ENSMUSG00000033307	Mif
ENSMUSG00000012519	Mlkl

ENSMUSG00000025355	Mmp19
ENSMUSG00000003348	Mob3a
ENSMUSG00000039616	Mocos
ENSMUSG00000062270	Morf4l1
ENSMUSG00000023861	Mpc1
ENSMUSG00000046805	Mpeg1
ENSMUSG00000039640	Mrpl12
ENSMUSG00000024436	Mrps18b
ENSMUSG00000029918	Mrps33
ENSMUSG00000038880	Mrps34
ENSMUSG00000024677	Ms4a6b
ENSMUSG00000079419	Ms4a6c
ENSMUSG00000024672	Ms4a7
ENSMUSG00000025044	Msr1
ENSMUSG00000075705	Msrb1
ENSMUSG00000005667	Mthfd2
ENSMUSG00000024300	Myo1f
ENSMUSG00000020437	Myo1g
ENSMUSG00000061315	Naca
ENSMUSG00000002204	Napsa
ENSMUSG00000027698	Nceh1
ENSMUSG00000015950	Ncf1
ENSMUSG00000026480	Ncf2
ENSMUSG00000022488	Nckap1l
ENSMUSG00000056234	Ncoa4
ENSMUSG00000020022	Ndufa12
ENSMUSG00000005510	Ndufs3
ENSMUSG00000047180	Neurl3
ENSMUSG00000058099	Nfam1
ENSMUSG00000032691	Nlrp3
ENSMUSG00000037601	Nme1
ENSMUSG00000056209	Npm3
ENSMUSG00000002108	Nr1h3
ENSMUSG00000026999	Nup35
ENSMUSG00000052776	Oas1a
ENSMUSG00000048779	P2ry6
ENSMUSG00000023249	Parp3
ENSMUSG00000022439	Parvg
ENSMUSG00000021496	Pcbd2
ENSMUSG00000027342	Pcna
ENSMUSG00000020553	Pctp

ENSMUSG00000011752	Pgam1
ENSMUSG00000062070	Pgk1
ENSMUSG00000038845	Phb
ENSMUSG00000025017	Pik3ap1
ENSMUSG00000039936	Pik3cd
ENSMUSG00000020901	Pik3r5
ENSMUSG00000021822	Plau
ENSMUSG00000046223	Plaur
ENSMUSG00000040061	Plcb2
ENSMUSG00000034330	Plcg2
ENSMUSG00000052160	Pld4
ENSMUSG00000020120	Plek
ENSMUSG00000031557	Plekha2
ENSMUSG00000026123	Plekhb2
ENSMUSG00000028494	Plin2
ENSMUSG00000031146	Plp2
ENSMUSG00000021871	Pnp
ENSMUSG00000071866	Ppia
ENSMUSG00000004455	Ppp1cc
ENSMUSG00000045679	Pqlc3
ENSMUSG00000028691	Prdx1
ENSMUSG00000067713	Prkag1
ENSMUSG00000026979	Psd4
ENSMUSG00000036835	Psenen
ENSMUSG00000060073	Psma3
ENSMUSG00000068749	Psma5
ENSMUSG00000069744	Psmb3
ENSMUSG00000022193	Psmb5
ENSMUSG00000024338	Psmb8
ENSMUSG00000079197	Psme2
ENSMUSG00000025429	Pstpip2
ENSMUSG00000056529	Ptafr
ENSMUSG00000043017	Ptgir
ENSMUSG00000004266	Ptpn6
ENSMUSG00000031506	Ptpn7
ENSMUSG00000026395	Ptprc
ENSMUSG00000030793	Pycard
ENSMUSG00000019832	Rab32
ENSMUSG00000033220	Rac2
ENSMUSG00000004952	Rasa4
ENSMUSG00000026430	Rassf5

ENSMUSG00000031167	Rbm3
ENSMUSG00000031387	Renbp
ENSMUSG00000026358	Rgs1
ENSMUSG00000030844	Rgs10
ENSMUSG00000031438	Rnf128
ENSMUSG00000030079	Ruvbl1
ENSMUSG00000019782	Rwdd1
ENSMUSG00000001020	S100a4
ENSMUSG00000001025	S100a6
ENSMUSG00000022876	Samsn1
ENSMUSG00000031101	Sash3
ENSMUSG00000078974	Sec61g
ENSMUSG00000028064	Sema4a
ENSMUSG00000074884	Serf2
ENSMUSG00000060147	Serpinb6a
ENSMUSG00000068856	Sf3b4
ENSMUSG00000028843	Sh3bgrl3
ENSMUSG00000054520	Sh3bp2
ENSMUSG00000059182	Skap2
ENSMUSG00000022372	Sla
ENSMUSG00000024737	Slc15a3
ENSMUSG00000025161	Slc16a3
ENSMUSG00000029175	Slc35f6
ENSMUSG00000032122	Slc37a2
ENSMUSG00000024327	Slc39a7
ENSMUSG00000034591	Slc41a2
ENSMUSG00000072620	Slfn2
ENSMUSG00000024217	Snrpc
ENSMUSG00000031662	Snx20
ENSMUSG00000029560	Snx8
ENSMUSG00000026222	Sp100
ENSMUSG00000070034	Sp110
ENSMUSG00000051457	Spn
ENSMUSG00000029304	Spp1
ENSMUSG00000024160	Spsb3
ENSMUSG00000034120	Srsf2
ENSMUSG00000032802	Srxn1
ENSMUSG00000028832	Stmn1
ENSMUSG00000020738	Sumo2
ENSMUSG00000021384	Susd3
ENSMUSG00000089847	Timm10b

ENSMUSG00000013701	Timm23
ENSMUSG00000033777	Tlr13
ENSMUSG00000091537	Tma7
ENSMUSG00000029390	Tmed2
ENSMUSG00000034947	Tmem106a
ENSMUSG00000038141	Tmem181a
ENSMUSG00000025521	Tmem192
ENSMUSG00000060538	Tmem219
ENSMUSG00000024401	Tnf
ENSMUSG00000028599	Tnfrsf1b
ENSMUSG00000045362	Tnfrsf26
ENSMUSG00000060126	Tpt1
ENSMUSG00000037318	Traf3ip3
ENSMUSG00000023992	Trem2
ENSMUSG00000018507	Trpv2
ENSMUSG00000041736	Tspo
ENSMUSG00000036918	Ttc7
ENSMUSG00000043091	Tuba1c
ENSMUSG00000073838	Tufm
ENSMUSG00000057130	Txnl4a
ENSMUSG00000030579	Tyrobp
ENSMUSG00000061613	U2af1
ENSMUSG00000026956	Uap1l1
ENSMUSG00000090137	Uba52
ENSMUSG00000027078	Ube2l6
ENSMUSG00000060860	Ube2s
ENSMUSG00000057948	Unc13d
ENSMUSG00000036908	Unc93b1
ENSMUSG00000020163	Uqcr11
ENSMUSG00000034116	Vav1
ENSMUSG00000028452	Vcp
ENSMUSG00000064090	Vrk2
ENSMUSG00000051506	Wdfy4
ENSMUSG00000058486	Wdr91
ENSMUSG00000024066	Xdh

Supplementary Table 4 Gene-Ontology (GO) functional enrichment analysis demonstrating that the defined set of 360 macrophage enriched transcripts are significantly enriched for immune-system related functions.

**Term**	**Count**	***P*-Value**	**Fold Enrichment**	**FDR**
GO:0006955: immune response	39	1.12E-13	4.18	1.88E-10
GO:0006954: inflammatory response	22	3.66E-09	4.94	6.15E-06
GO:0009611: response to wounding	26	2.34E-08	3.78	3.94E-05
GO:0006935: chemotaxis	19	3.86E-12	8.81	6.50E-09
GO:0048584: positive regulation of response to stimulus	17	8.45E-07	4.62	0.00142109
GO:0002709: regulation of T cell mediated immunity	7	8.47E-07	19.64	0.00142439
GO:0002703: regulation of leukocyte mediated immunity	11	1.05E-06	7.94	0.00177359
GO:0002819: regulation of adaptive immune response	10	1.33E-06	9.02	0.00223765
GO:0002706: regulation of lymphocyte mediated immunity	10	4.81E-06	7.77	0.00808705
